# Boosting Higgs pair production in the $b\bar{b}b\bar{b}$ final state with multivariate techniques

**DOI:** 10.1140/epjc/s10052-016-4215-5

**Published:** 2016-07-08

**Authors:** J. Katharina Behr, Daniela Bortoletto, James A. Frost, Nathan P. Hartland, Cigdem Issever, Juan Rojo

**Affiliations:** Physics Department, University of Oxford, 1 Keble Road, Oxford, UK

## Abstract

The measurement of Higgs pair production will be a cornerstone of the LHC program in the coming years. Double Higgs production provides a crucial window upon the mechanism of electroweak symmetry breaking and has a unique sensitivity to the Higgs trilinear coupling. We study the feasibility of a measurement of Higgs pair production in the $b\bar{b}b\bar{b}$ final state at the LHC. Our analysis is based on a combination of traditional cut-based methods with state-of-the-art multivariate techniques. We account for all relevant backgrounds, including the contributions from light and charm jet mis-identification, which are ultimately comparable in size to the irreducible 4*b* QCD background. We demonstrate the robustness of our analysis strategy in a high pileup environment. For an integrated luminosity of ${\mathcal {L}}=3$ ab$^{-1}$, a signal significance of $S/\sqrt{B}\simeq 3$ is obtained, indicating that the $b\bar{b}b\bar{b}$ final state alone could allow for the observation of double Higgs production at the High Luminosity LHC.

## Introduction

The measurement of double Higgs production will be one of the central physics goals of the LHC program in its recently started high-energy phase, as well as for its future high-luminosity upgrade (HL-LHC) which aims to accumulate a total integrated luminosity of 3 ab$^{-1}$ [1, 2]. Higgs pair production [3] is directly sensitive to the Higgs trilinear coupling $\lambda$ and provides crucial information on the electroweak symmetry breaking mechanism. It also probes the underlying strength of the Higgs interactions at high energies, and it can be used to test the composite nature of the Higgs boson [4, 5]. While Standard Model (SM) cross sections are small, many Beyond the SM (BSM) scenarios predict enhanced rates for double Higgs production; therefore searches have already been performed by ATLAS and CMS with Run I data [6–10] and will continue at Run II. The study of Higgs pair production will also be relevant to any future high-energy collider, either at a 100 TeV circular machine [11–14] or at a linear or circular electron–positron collider [15].

Analogously to single Higgs production [16], in the SM the dominant mechanism for the production of a pair of Higgs bosons at the LHC is gluon fusion (see [3, 17] and references therein). For a center-of-mass energy of $\sqrt{s} = 14$ TeV, the next-to-next-to-leading order (NNLO) total cross section is approximately 40 fb [18], which is increased by a further few percent once next-to-next-to-leading logarithmic (NNLL) corrections are accounted for [19]. Feasibility studies in the case of a SM-like Higgs boson in the gluon-fusion channel at the LHC have been performed for different final states, including $b\bar{b}\gamma \gamma$ [20–22], $b\bar{b}\tau ^+\tau ^-$ [23–26], $b\bar{b}W^+W^-$ [25, 27] and $b\bar{b}b\bar{b}$ [21, 23, 25, 28, 29]. While these studies differ in their quantitative conclusions, the consistent picture emerges that the ultimate precision in the determination of the Higgs trilinear coupling $\lambda$ requires the full integrated luminosity of the HL-LHC, ${\mathcal {L}}=3$ ab$^{-1}$ and should rely on the combination of different final states. The interplay between kinematic distributions for the extraction of $\lambda$ from the measured cross sections and the role of the associated theoretical uncertainties have been intensely scrutinised recently [17, 30–37].

In addition to the gluon-fusion channel, Higgs pairs can also be produced in the vector-boson fusion channel *hhjj* [5, 26, 38, 39], the associated production modes *hhW* and *hhZ* [3, 40, 41] (also known as Higgs-Strahlung), and also in association with top quark pairs $hht\bar{t}$ [42]. All these channels are challenging due to the small production rates: at 14 TeV, the inclusive total cross sections are 2.0 fb for VBF *hhjj* [43], 0.5 fb for *W*(*Z*)*hh* [3] and 1.0 for $hht\bar{t}$ [42].

While the SM production rates for Higgs pairs are small, they are substantially enhanced in a variety of BSM scenarios. Feasibility studies of Higgs pair production in New Physics models have been performed in a number of different frameworks, including Effective Field Theories (EFTs) with higher-dimensional operators and anomalous Higgs couplings [14, 44–50], resonant production in models such as extra dimensions [51–54], and Supersymmetry and Two Higgs Doublet models (2HDMs) [55–61]. Since BSM dynamics modify the kinematic distributions of the Higgs decay products, for instance boosting the di-Higgs system, different analysis strategies might be required for BSM Higgs pair searches as compared to SM measurements.

Searches for the production of Higgs pairs have already been performed with 8 TeV Run I data by ATLAS in the $b\bar{b}b\bar{b}$ [7] and $b\bar{b}\gamma \gamma$ [8] final states, and by CMS in the same $b\bar{b}b\bar{b}$ [9] and $b\bar{b}\gamma \gamma$ [10] final states. In addition, ATLAS has presented [6] a combination of its di-Higgs searches in the $bb\tau \tau ,$ $\gamma \gamma WW^*$, $\gamma \gamma bb$ and *bbbb* final states. Many other exotic searches involve Higgs pairs in the final state, such as the recent search for heavy Higgs bosons *H* [62].

In the context of SM production, the main advantage of the $b\bar{b}b\bar{b}$ final state is the enhancement of the signal yield from the large branching fraction of Higgs bosons into $b\bar{b}$ pairs, $\mathrm{BR}\left( H\rightarrow b\bar{b}\right) \simeq 0.57$ [16]. However, a measurement in this channel needs to deal with an overwhelming QCD multi-jet background. Recent studies of Higgs pair production in this final state [28, 29] estimate that, for an integrated luminosity of ${{\mathcal {L}}}=3$ ab$^{-1}$, a signal significance of around $S/\sqrt{B}\simeq 2.0$ can be obtained. In these analysis, irreducible backgrounds such as 4*b* and $t\bar{t}$ are included, however, the reducible components, in particular *bbjj* and *jjjj*, are neglected. These can contribute to the signal yield when light and charm jets are mis-identified as *b*-jets. Indeed, due to both selection effects and *b*-quark radiation in the parton shower, the contribution of the 2*b*2*j* process is as significant as the irreducible 4*b* component.

In this work, we revisit the feasibility of SM Higgs pair production by gluon fusion in the $b\bar{b}b\bar{b}$ final state at the LHC. Our strategy is based upon a combination of traditional cut-based methods and multivariate analysis (MVA). We account for all relevant backgrounds, including the contribution from mis-identified light and charm jets. We also assess the robustness of our analysis strategy in an environment with high pileup (PU). Our results indicate that the $b\bar{b}b\bar{b}$ final state alone should allow for the observation of double Higgs production at the HL-LHC.

The structure of this paper proceeds as follows. In Sect. 2 we present the modeling of the signal and background processes with Monte Carlo event generators. In Sect. 3 we introduce our analysis strategy, in particular the classification of individual events into different categories according to their topology. Results of the cut-based analysis are then presented in Sect. 4. In Sect. 5 we illustrate the enhancement of signal significance using multivariate techniques, and we assess the robustness of our results against the effects of PU. In Sect. 6 we conclude and outline future studies to estimate the accuracy in the determination of the trilinear coupling $\lambda$ and to provide constraints in BSM scenarios.

## Modeling of signal and background processes

In this section we discuss the Monte Carlo generation of the signal and background process samples used in this analysis. We shall also discuss the modeling of detector resolution effects.

### Higgs pair production in gluon fusion

Higgs pair production is simulated at leading order (LO) using MadGraph5_aMC@NLO [63]. We use a tailored model [34] for gluon-fusion Higgs boson pair production which includes mass effects from the exact form factors for the top-quark triangle and box loops [64]. Equivalent results can be obtained using the recently available functionalities for the calculation of loop-induced processes [65] in MadGraph5_aMC@NLO. The calculation is performed in the $n_f=4$ scheme, accounting for *b*-quark mass effects. The renormalisation and factorisation scales are taken to be $\mu _F=\mu _R=H_T/2$, with

1 $$\begin{aligned} H_T\equiv \sum _i \sqrt{p_{T,i}^2+m_i^2}, \end{aligned}$$

the scalar sum of the transverse masses of all final-state particles. For the input parton distribution functions (PDFs) we adopt the NNPDF 3.0 $n_f=4$ LO set [66] with $\alpha _s(m_Z^2)=0.118$, interfaced via LHAPDF6 [67]. The Higgs boson couplings and branching ratios are set to their SM values, and its mass is taken to be $m_h=125$ GeV [68–70]. In the SM, the Higgs trilinear coupling is given by $\lambda =m_h^2/2v^2$, with $v\simeq 246$ GeV the Higgs vacuum expectation value.

**Fig. 1:**
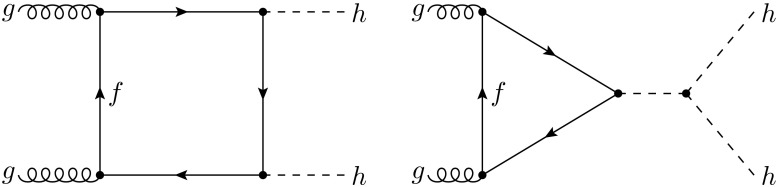
Representative Feynman diagrams for Higgs pair production in gluon fusion at leading order. Only the fermion triangle loop diagram (*right*) is directly sensitive to the Higgs trilinear coupling $\lambda$. In the SM, the fermion loops are dominated by the contribution from the top quark

**Table 1:** Details of the signal and background Monte Carlo samples used in this work. Also provided are the inclusive *K*-factors which are applied to reproduce the known higher-order results

Process	Generator	$N_{\mathrm {evt}}$	$\sigma _{\mathrm {LO}}$ (pb)	*K*-factor
$pp \rightarrow hh\rightarrow 4b$	MadGraph5_aMC@NLO	1M	$6.2\times 10^{-3}$	2.4 (NNLO+NNLL [18, 19])
$pp \rightarrow b\bar{b}b\bar{b}$	SHERPA	3M	$1.1\times 10^3$	1.6 (NLO [63])
$pp \rightarrow b\bar{b}jj$	SHERPA	3M	$2.7\times 10^5$	1.3 (NLO [63])
$pp \rightarrow jjjj$	SHERPA	3M	$9.7\times 10^6$	0.6 (NLO [77])
$pp \rightarrow t\bar{t}\rightarrow b\bar{b}jjjj$	SHERPA	3M	$2.5\times 10^3$	1.4 (NNLO+NNLL [78])

In Fig. 1 we show representative Feynman diagrams for LO Higgs pair production in gluon fusion. The non-trivial interplay between the heavy quark box and the triangle loop diagrams can lead to either constructive or destructive interference and complicates the extraction of the trilinear coupling $\lambda$ from the measurement of the Higgs pair production cross section. Higher-order corrections [17, 18] are dominated by gluon radiation from either the initial-state gluons or from the heavy quark loops.

The total inclusive cross section for this processes is known up to NNLO [18]. Resummed NNLO+NNLL calculations for Higgs pair production are also available [19], leading to a moderate enhancement of the order of few percent as compared to the fixed-order NNLO calculation. To achieve the correct higher-order value of the integrated cross section, we rescale our LO signal sample to match the NNLO+NNLL inclusive calculation. This corresponds to a *K*-factor $\sigma _\mathrm{NNLO+NNLL}/\sigma _\mathrm{LO}=2.4$, as indicated in Table 1.

Parton-level signal events are then showered with the Pythia8 Monte Carlo [71, 72], version v8.201. We use the default settings for the modeling of the underlying event (UE), multiple parton interactions (MPI), and PU, by means of the Monash 2013 tune [73], based on the NNPDF2.3LO PDF set [74, 75].

### Backgrounds

Background samples are generated at leading order with SHERPA [76] v2.1.1. As in the case of the signal generation, the NNPDF 3.0 $n_f = 4$ LO set with strong coupling $\alpha _s(m_Z^2)=0.118$ is used for all samples, and we use as factorisation and renormalisation scales $\mu _F=\mu _R=H_T/2$. We account for all relevant background processes that can mimic the $hh\rightarrow 4b$ signal process. This includes QCD 4*b* multi-jet production, as well as QCD 2*b*2*j* and 4*j* production, and top-quark pair production. The latter is restricted to the fully hadronic final state, since leptonic decays of top quarks can be removed by requiring a lepton veto. Single Higgs production processes such as $Z(\rightarrow b\bar{b})h(\rightarrow b\bar{b})$ and $t\bar{t}h(\rightarrow b\bar{b})$ (see Appendix A) along with electroweak backgrounds e.g. $Z(\rightarrow b\bar{b})b\bar{b}$, are much smaller than the QCD backgrounds [28, 29] and are therefore not included in the present analysis.

The LO cross sections for the background samples have been rescaled so that the integrated distributions reproduce known higher-order QCD results. For the 4*j* sample, we rescale the LO cross section using the BLACKHAT [77] calculation, resulting in an NLO/LO *K*-factor of 0.6. For the 4*b* and 2*b*2*j* samples NLO/LO *K*-factors of 1.6 and 1.3, respectively, have been determined using MadGraph5_aMC@NLO [63]. Finally, the LO cross section for $t\bar{t}$ production has been rescaled to match the NNLO+NNLL calculation of Ref. [78], leading to a *K*-factor of 1.4. The *K*-factors that we use to rescale the signal and background samples are summarised in Table 1.

At the generation level, the following loose selection cuts are applied to background events. Each final-state particle in the hard process must have $p_T \ge 20$ GeV, and be located in the central rapidity region with $| \eta | \le 3.0$. At the matrix-element level all final-state particles must also be separated by a minimum $\Delta R_{\mathrm {min}} =0.1$. We have checked that these generator-level cuts are loose enough to have no influence over the analysis cuts. From Table 1 we see that the $t\bar{t}$ and QCD 4*b* cross sections are of the same order of magnitude. However, the former can be efficiently reduced by using top quark reconstruction criteria. The *bbjj* cross section is more than two orders of magnitude larger than the 4*b* result, but it will be suppressed by the light and charm jet mis-identification rates, required to contribute to the 4*b* final state.

As a cross-check of the SHERPA background cross sections reported in Table 1, we have produced leading-order multi-jet samples using MadGraph5_aMC@NLO, benchmarked with the results for the same processes reported in Ref. [63]. Using common settings, we find agreement, within scale uncertainties, between the MadGraph5_aMC@NLO and SHERPA calculations of the multi-jet backgrounds.

### Modeling of detector resolution

While it is beyond the scope of this work to perform a full detector simulation, it is important to include an estimate of detector effects in the analysis, particularly for the finite energy and angular resolutions which directly degrade the reconstruction of important kinematic variables, such as the invariant mass of the Higgs candidates. Here we simulate the finite energy resolution of the ATLAS and CMS hadronic calorimeters by applying a Gaussian smearing of the transverse momentum $p_T$ with mean zero and standard deviation $\sigma _E$ for all final-state particles before jet clustering, that is,

2 $$\begin{aligned} p_T^{(i)} \, \rightarrow \, p_T^{(i)\prime }= \left( 1+ r_i\cdot \sigma _E \right) \, p_T^{(i)}, \quad i=1,\ldots ,N_\mathrm{part}, \end{aligned}$$

with $r_i$ a univariate Gaussian random number, different for each of the $N_\mathrm{part}$ particles in the event. We take as a baseline value for the transverse-momentum smearing a factor of $\sigma _E=5~\%$.

To account for the finite angular resolution of the calorimeter, the $\left( \eta ,\phi \right)$ plane is divided into regions of $\Delta \eta \times \Delta \phi =0.1\times 0.1$, and each final-state particle which falls in each of these cells is set to the same $\eta$ and $\phi$ values of the center of the corresponding cell. Finally, the energy of each final-state particle is recalculated from the smeared $p_T^\prime$, $\eta ^\prime$ and $\phi ^\prime$ values to ensure that the resulting four-momentum is that of a light-like particle, since we neglect all jet constituent masses in this analysis.

Our modeling of detector simulation has been tuned to lead to a mass resolution of the reconstructed Higgs candidates consistent with the hadronic mass resolutions of the ATLAS and CMS detectors [79–81], as discussed in Sect. 3.5.

## Analysis strategy

In this section we describe our analysis strategy. First of all we discuss the settings for jet clustering and the strategy for jet *b*-tagging. Following this we discuss the categorisation of events into different topologies, and how the different topologies may be prioritised. We motivate our choice of analysis cuts by comparing signal and background distributions for representative kinematic variables. Finally, we describe the simulation of PU and validate the PU-subtraction strategy.

### Jet reconstruction

After the parton shower, final-state particles are clustered using the jet reconstruction algorithms of FastJet [82, 83], v3.1.0. Here we use the following jet definitions:

- *Small-R jets*. These are jets reconstructed with the anti-$k_T$ clustering algorithm [84] with $R=0.4$ radius. These small-*R* jets are required to have transverse momentum $p_T \ge 40$ GeV and pseudo-rapidity $|\eta |<2.5$, within the central acceptance of ATLAS and CMS, and therefore within the region where *b*-tagging is possible.
- *Large-R jets*. These jets are also constructed with the anti-$k_T$ clustering algorithm, now using a $R=1.0$ radius. Large-*R* jets are required to have $p_T \ge 200$ GeV and lie in a pseudo-rapidity region of $|\eta |<2.0$. The more restrictive range in pseudo-rapidity as compared to the small-*R* jets is motivated by mimicking the experimental requirements in ATLAS and CMS related to the track-jet based calibration [85, 86]. In addition to the basic $p_T$ and $\eta$ acceptance requirements, large-*R* jets should also satisfy the BDRS mass-drop tagger (MDT) [87] conditions, where the FastJet default parameters of $\mu _\mathrm{mdt} = 0.67$ and $y_\mathrm{mdt}=0.09$ are used. Before applying the BDRS tagger, the large-*R* jet constituents are reclustered with the Cambridge/Aachen (C/A) algorithm [88, 89] with $R=1.0$. In the case of the analysis including PU, a trimming algorithm [106] is applied to all large-*R* jets to mitigate the effects of PU, especially on the jet mass. For further details, see Sect. 3.5.
- *Small-R subjets*. All final-state particles are clustered using the anti-$k_T$ algorithm, but this time with a smaller radius parameter, namely $R=0.3$. The resulting anti-$k_T$ $R=0.3$ (AKT03) jets are then ghost-associated to each large-*R* jets in order to define its subjets [7]. These AKT03 subjets are required to satisfy $p_T > 50$ GeV and $|\eta |<2.5$, and they will be the main input for *b*-tagging in the boosted category.

For the boosted and intermediate categories, which involve the use of large-*R* jets, we use jet substructure variables [90, 91] to improve the significance of the discrimination between signal and background events in the MVA. In particular we consider the following substructure variables:

- The $k_T$-splitting scale [87, 92]. This variable is obtained by reclustering the constituents of a jet with the $k_T$ algorithm [93], which usually clusters last the harder constituents, and then taking the $k_T$ distance measure between the two subjets at the final stage of the recombination procedure,
  3 $$\begin{aligned} \sqrt{d_{12}} \equiv \mathrm{min}\left( p_{T,1},p_{T,2}\right) \cdot \Delta R_{12}. \end{aligned}$$
  with $p_{T,1}$ and $p_{T,2}$ the transverse momenta of the two subjets merged in the final step of the clustering, and $\Delta R_{12}$ the corresponding angular separation.
- The ratio of 2-to-1 subjettiness $\tau _{21}$ [94, 95]. The *N*-subjettiness variables $\tau _N$ are defined by clustering the constituents of a jet with the exclusive $k_t$ algorithm [96] and requiring that *N* subjets are found,
  4 $$\begin{aligned} \tau _N\equiv & {} \frac{1}{d_0} \sum _k p_{T,k}\cdot \mathrm{min}\left( \delta R_{1k}, \ldots , \delta R_{Nk}\right) ,\nonumber \\ d_0\equiv & {} \sum _k p_{T,k}\cdot R, \end{aligned}$$
  where $p_{T,k}$ is the $p_T$ of the constituent particle *k* and $\delta R_{ik}$ the distance from subjet *i* to constituent *k*. In this work we use as input to the MVA the ratio of 2-subjettiness to 1-subjettiness, namely
  5 $$\begin{aligned} \tau _{21} \equiv \frac{\tau _2}{\tau _1}, \end{aligned}$$
  which provides good discrimination between QCD jets and jets arising from the decay of a heavy resonance.
- The ratios of energy correlation functions (ECFs) $C^{(\beta )}_2$ [97] and $D_2^{(\beta )}$ [98]. The ratio of energy correlation functions $C_2^{(\beta )}$ is defined as
  6 $$\begin{aligned} C_2^{(\beta )} \equiv \frac{ \mathrm{ECF}(3,\beta ) \mathrm{ECF}(1,\beta )}{\left[ \mathrm{ECF}(2,\beta )\right] ^2}, \end{aligned}$$
  while $D_2^{(\beta )}$ is instead defined as a double ratio of ECFs, that is,
  7 $$\begin{aligned}&e_3^{(\beta )}\equiv \frac{ \mathrm{ECF}(3,\beta )}{\left[ \mathrm{ECF}(1,\beta )\right] ^3}, \quad e_2^{(\beta )}\equiv \frac{ \mathrm{ECF}(2,\beta )}{\left[ \mathrm{ECF}(1,\beta )\right] ^2},\nonumber \\&\quad D_2^{(\beta )} \equiv \frac{ e_3^{(\beta )}}{\left( e_2^{(\beta )} \right) ^3}. \end{aligned}$$
  The energy correlation functions $\mathrm{ECF}(N,\beta )$ are defined in [97] with the motivation that $(N+1)$-point correlators are sensitive to *N*-prong substructure. The free parameter $\beta$ is set to a value of $\beta =2$, as recommended by Refs. [97, 98].

### Tagging of *b*-jets

In this analysis we adopt a *b*-tagging strategy along the lines of current ATLAS performance [91, 99], though differences with respect to the corresponding CMS settings [100, 101] do not modify qualitatively our results. For each jet definition described above, a different *b*-tagging strategy is adopted:

- *Small-R jets*. If a small-*R* jet has at least one *b*-quark among their constituents, it will be tagged as a *b*-jet with probability $f_b$. In order to be considered in the *b*-tagging algorithm, *b*-quarks inside the small-*R* jet should satisfy $p_T \ge 15$ GeV [99]. The probability of tagging a jet is not modified if more than one *b*-quark is found among the jet constituents. If no *b*-quarks are found among the constituents of this jet, it can be still be tagged as a *b*-jet with a mistag rate of $f_l$, unless a charm quark is present instead, and in this case the mistag rate is $f_c$. Only jets that contain at least one (light or charm) constituent with $p_T \ge 15$ GeV can induce a fake *b*-tag. We attempt to *b*-tag only the four (two) hardest small-*R* jets in the resolved (intermediate) category. Attempting to *b*-tag all of the small-*R* jets that satisfy the acceptance cuts worsens the overall performance as the rate of fake *b*-tags increases substantially.
- *Large-R jets*. Large-*R* jets are *b*-tagged by ghost-associating anti-$k_T$ $R=0.3$ (AKT03) subjets to the original large-*R* jets [7, 91, 102, 103]. A large-*R* jet is considered *b*-tagged if both the leading and the subleading AKT03 subjets, where the ordering is done in the subjet $p_T$, are both individually *b*-tagged, with the same criteria as the small-*R* jets. Therefore, a large-*R* jet where the two leading subjets have at least one *b*-quark will be tagged with probability $f_b^2$. As in the case of small-*R* jets, we only attempt to *b*-tag the two leading subjets, else one finds a degradation of the signal significance. The treatment of the *b*-jet mis-identification from light and charm jets is the same as for the small-*R* jets.

For the *b*-tagging probability $f_b$, along with the *b*-mistag probability of light ($f_l$) and charm ($f_c$) jets, we use the values $f_b=0.8$, $f_l=0.01$ and $f_c=0.1$.

**Fig. 2:**
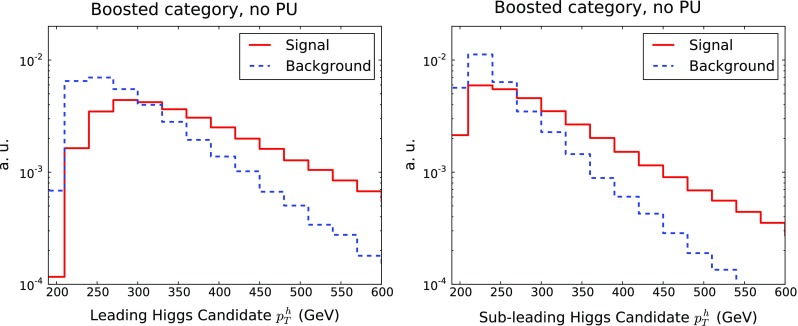
Comparison of the $p_T$ distributions of the leading (*left*) and subleading (*right*) large-*R* jets in the boosted category, for signal and background events. Distributions have been normalised to unity. The total background is the sum of all components listed in Table 1

### Event categorisation

The present analysis follows a strategy similar to the scale-invariant resonance tagging of Ref. [51]. Rather than restricting ourselves to a specific event topology, we aim to consistently combine the information from the three possible topologies: boosted, intermediate and resolved, with the optimal cuts for each category being determined separately. This approach is robust under variations of the underlying production model of Higgs pairs, for instance in the case of BSM dynamics, which can substantially increase the degree of boost in the final state.

The three categories are defined as follows:

- *Boosted category*. An event which contains at least two large-*R* jets, with the two leading jets being *b*-tagged. Each of these two *b*-tagged, large-*R* jets are therefore candidates to contain the decay products of a Higgs boson.
- *Intermediate category*. An event with exactly one *b*-tagged, large-*R* jet, which is assigned to be the leading Higgs candidate. In addition, we require at least two *b*-tagged, small-*R* jets, which must be separated with respect to the large-*R* jet by an angular distance of $\Delta R\ge 1.2$. The subleading Higgs boson candidate is reconstructed by selecting the two *b*-tagged small-*R* jets that minimise the difference between the invariant mass of the large-*R* jet with that of the dijet obtained from the sum of the two small-*R* jets.
- *Resolved category*. An event with at least four *b*-tagged small-*R* jets. The two Higgs candidates are reconstructed out of the leading four small-*R* jets in the event by considering all possible combinations of forming two pairs of jets and then choosing the configuration that minimises the relative difference of dijet masses.

**Fig. 3:**
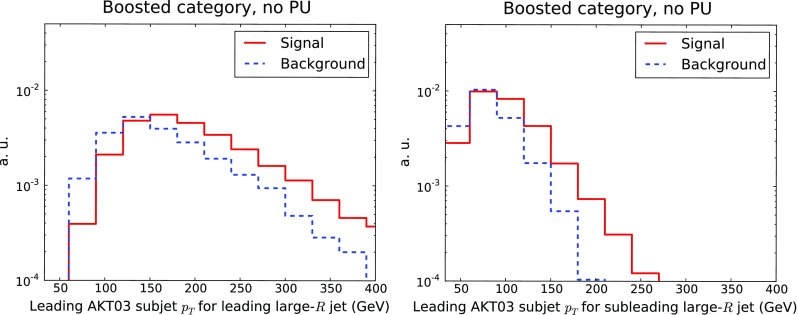
Same as Fig. 2 for the leading (*left*) and subleading (*right*) AKT03 subjets in the subleading Higgs candidate large-*R* jet

**Fig. 4:**
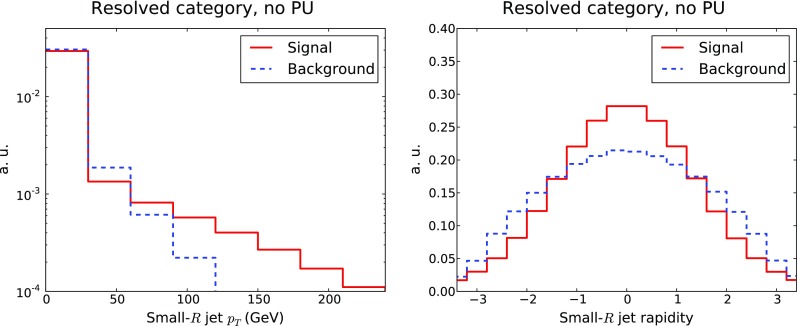
Same as Fig. 2, now for the $p_T$ and rapidity distributions of the small-*R* jets corresponding to the resolved selection

Once a Higgs boson candidate has been identified, its invariant mass is required to lie within a fixed window of width $80~\mathrm{GeV}$ around the nominal Higgs boson mass of $m_h= 125$ GeV. Specifically we require the condition

8 $$\begin{aligned} |m_{h,j} - 125~\mathrm{GeV}| < 40~\mathrm{GeV},\quad j=1,2, \end{aligned}$$

where $m_{h,j}$ is the invariant mass of each of the two reconstructed Higgs candidates. This cut is substantially looser than the corresponding cut used in the typical ATLAS and CMS $h\rightarrow b\bar{b}$ analyses [79, 80]. The motivation for such a loose cut is that further improvements of the signal significance will be obtained using an MVA. Only events where the two Higgs candidates satisfy Eq. (8) are classified as signal events.

These three categories are not exclusive: a given event can be assigned to more than one category, for example, satisfying the requirements of both the intermediate and the resolved categories at the same time. The exception is the boosted and intermediate categories, which have conflicting jet selection requirements.

This is achieved as follows. First of all we perform an inclusive analysis, and optimise the signal significance $S/\sqrt{B}$ in each of the three categories separately, including the MVA. We find that the category with highest significance is the boosted one, followed by the intermediate and the resolved topologies, the latter two with similar significance. Therefore, when ascertaining in which category an event is to be exclusively placed: if the event satisfies the boosted requirements, it is assigned to this category, else we check if it suits the intermediate requirements. If the event also fails the intermediate category requirements, we then check if it passes the resolved selection criteria. The resulting exclusive event samples are then separately processed through the MVA, allowing for a consistent combination of the significance of the three event categories.

### Motivation for basic kinematic cuts

We now motivate the kinematic cuts applied to the different categories, comparing representative kinematic distributions between signal and background events. First of all, we present results without PU, and then discuss the impact of PU on the description of the kinematic distributions. In the following, all distributions are normalised to their total integral.

In Fig. 2 we show the $p_T$ distributions of the leading and subleading large-*R* jets in the boosted category. We observe that the background distribution falls off more rapidly as a function of $p_T$ than the di-Higgs signal. On the other hand, the cut in $p_T$ cannot be too strong to avoid a substantial degradation of signal selection efficiency, specially taking into account the subleading large-*R* jet. This comparison justifies the cut of $p_T \ge 200$ GeV for the large-*R* jets that we impose in the boosted category.

Another selection requirement for the boosted category is that the two leading AKT03 subjets of the large-*R* jet should satisfy $p_T \ge 50$ GeV. To motivate this cut, in Fig. 3 we show the distribution in $p_T$ of the leading and subleading AKT03 subjets in the subleading large-*R* jet in events corresponding to the boosted category. It is clear from the comparison that the subjet $p_T$ spectrum is relatively harder in the signal with respect to the background. On the other hand, considering the subleading AKT03 subjet, this cut in $p_T$ cannot be too harsh to maintain a high signal selection efficiency. Therefore, as for the previous distribution, the chosen cut value is a compromise between suppressing backgrounds but keeping a large fraction of signal events is crucial.

**Fig. 5:**
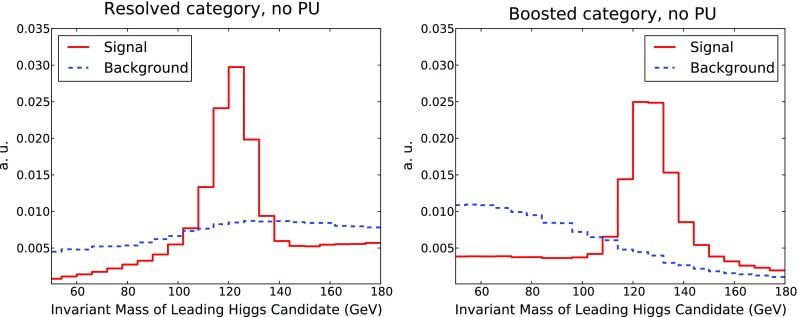
Same as Fig. 2 for the invariant mass distribution of the leading Higgs candidates in the resolved (*left*) and boosted (*right*) selections

**Fig. 6:**
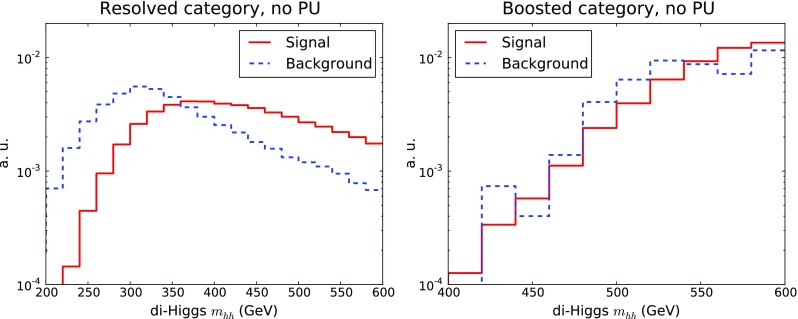
Same as Fig. 2 for the invariant mass distribution of the di-Higgs system $m_{hh}$, in the resolved (*left*) and boosted (*right*) categories

Turning to the resolved category, an important aspect to account for in the selection cuts is the fact that the $p_T$ distribution of the four leading small-*R* jets of the event can be relatively soft, especially for the subleading jets. As noted in [29], this is due to the fact that the boost from the Higgs decay is moderate; therefore the $p_T$ selection cuts for the small-*R* jets cannot be too large. In Fig. 4 we show the distribution in $p_T$ of the four leading small-*R* jets in signal and background events: we observe that both distributions peak at $p_T \le 50$ GeV, with the signal distribution falling off less steeply at large $p_T$. The feasibility of triggering on four small-*R* jets with a relatively soft $p_T$ distribution is one of the experimental challenges for exploiting the resolved category in this final state, and hence the requirement that $p_T \ge 40$ GeV for the small-*R* jets. In Fig. 4 we also show the rapidity distribution of the small-*R* jets in the resolved category. As expected, the production is mostly central, and more so in the case of signal events, since backgrounds are dominated by QCD *t*-channel exchange; therefore the selection criteria on the jet rapidity are very efficient.

One of the most discriminating selection cuts is the requirement that the invariant mass of the Higgs candidate (di)jets must lie within a window around the nominal Higgs value, Eq. (8). In Fig. 5 we show the invariant mass of the leading reconstructed Higgs candidates, before the Higgs mass window selection is applied, for the resolved and boosted categories. While the signal distribution naturally peaks at the nominal Higgs mass, the background distributions show no particular structure. The width of the Higgs mass peak is driven both from QCD effects, such as initial-state radiation (ISR) and out-of-cone radiation, as well as from the four-momentum smearing applied to final-state particles as part of our minimal detector simulation.

The invariant mass of the di-Higgs system is another important kinematic distribution for this process. The di-Higgs invariant mass is a direct measure of the boost of the system, which in BSM scenarios can be substantially enhanced, for instance due to specific $d=6$ EFT operators [14]. One important advantage of the $b\bar{b}b\bar{b}$ final state for di-Higgs production is that it significantly increases the reach in $m_{hh}$ as compared to other channels with smaller branching ratios, such as $2b2\gamma$ or $2b2\tau$. In Fig. 6 we show the invariant mass distribution of the reconstructed Higgs pairs, comparing the resolved and the boosted categories.

**Fig. 7:**
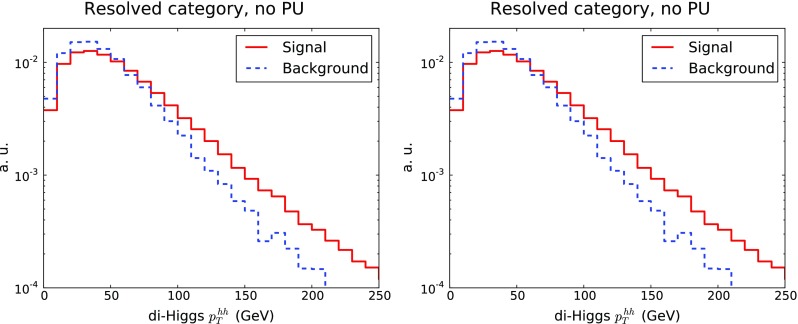
Same as Fig. 2 for the transverse momentum distribution of the di-Higgs system $p_T^{hh}$

In the resolved case, we see that the distribution in $m_{hh}$ is rather harder for the signal as compared to the background, and therefore one expects that cutting in $m_{hh}$ would help signal discrimination. For the boosted category the overall trend of the $m_{hh}$ distribution is different because of the selection criteria, and the distribution now peaks at higher values of the invariant mass. In this case, signal and background distributions are not significantly differentiated. Note that at parton level the $m_{hh}$ distribution for signal events has a kinematic cut-off at $m_{hh}^\mathrm{min}=250$ GeV, which is smeared due to parton shower and detector resolution effects.

**Fig. 8:**
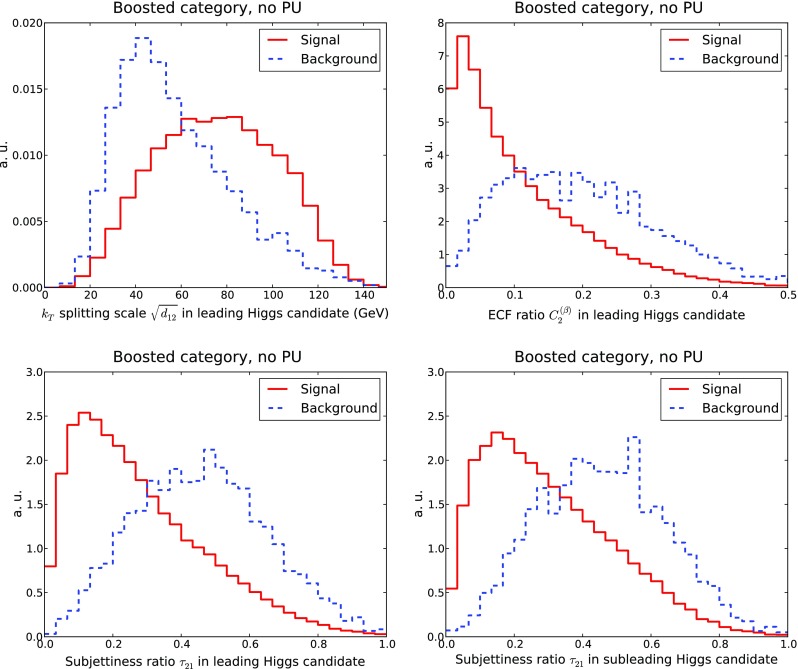
Distribution of representative substructure variables in the boosted category at the end of the cut-based analysis, to be used as input to the MVA. From *top to bottom* and from *left to right* we show the $k_t$ splitting scale $\sqrt{d_{12}}$, the energy correlation ratio $C_2^{(\beta )}$ and the subjettiness ratio $\tau _{21}$ for the leading Higgs. In the case of $\tau _{21}$ the distributions for the subleading Higgs are also given

In Fig. 7 we show the transverse momentum of the di-Higgs system, $p_T^{hh}$, for the resolved and boosted categories. Once more we see that the background has a steeper fall-off in $p_T^{hh}$ than the signal, in both categories, therefore this variable should provide additional discrimination power, motivating its inclusion as one of the inputs for the MVA. In our LO simulation the $p_T^{hh}$ distribution is generated by the parton shower, an improved theoretical description would require merging higher-multiplicity matrix elements [35] or matching to the NLO calculation [17],

We shall now investigate the discrimination power provided by jet substructure quantities. In Fig. 8 we show the distributions of representative substructure variables for the boosted category: the $k_T$ splitting scale $\sqrt{d_{12}}$, Eq. (3), the ECF ratio $C_2^{(\beta )}$, Eq. (6), and the 2–to–1 subjettiness ratio $\tau _{21}$, Eq. (5), all for the leading Higgs candidates, and also $\tau _{21}$ for the subleading Higgs candidates.

From Fig. 8 we observe how for these substructure variables the shapes of the signal and background distributions reflect the inherent differences in the internal structure of QCD jets and jets originating from Higgs decays. Signal and background distributions peak in rather different regions. For example, the $k_t$ splitting scale $\sqrt{d_{12}}$ peaks around 80 GeV (40 GeV) for signal (background) events, while the distribution of the ECF ratio $C_2^{(\beta )}$ is concentrated at small values for signal and is much broader for background events. From Fig. 8 we also see the distributions of the subjettiness ratio $\tau _{21}$ are reasonably similar for both the leading and the subleading jets.

### Impact of pileup

Now we turn to discuss how the description of kinematic distributions for signal and background processes are modified in the presence of pileup. To study the impact of PU, Minimum Bias events have been generated with Pythia8, and then superimposed to the signal and background samples described in Sect. 2. We have explored two scenarios, one with a number of PU vertices per bunch crossing of $n_\mathrm{PU}=80$, and another with $n_\mathrm{PU}=150$. In the following we adopt $n_\mathrm{PU}=80$ as our baseline, and denote this scenario by PU80. We have verified that the combined signal significance is similar if $n_\mathrm{PU}=150$ is adopted instead.

In order to subtract PU in hadronic collisions, a number of techniques are available [87, 102, 104–114].^1^ In this work, PU is subtracted with the SoftKiller (SK) method [111], as implemented in FastJet, whose performance has been shown to improve the commonly used area-based subtraction [104]. The idea underlying SoftKiller consists of eliminating particles below a given cut-off in their transverse momentum, $p_T^\mathrm{(cut)}$, whose value is dynamically determined so that the event-wide transverse-momentum flow density $\rho$ vanishes, where $\rho$ is defined as

9 $$\begin{aligned} \rho \equiv \mathrm{median}_i \Bigg \{ \frac{p_{Ti}}{A_i}\Bigg \}, \end{aligned}$$

and where the median is computed over all the regions *i* with area $A_i$ and transverse momentum $p_{Ti}$ in which the $\left( \eta ,\phi \right)$ plane is partitioned.

From its definition in terms of the median, it follows that the value of $p_T^{(\mathrm cut)}$ will be dynamically raised until half of the regions have $p_{Ti}=0$. The size and number of these regions is a free parameter of the algorithm—here we will use square regions with length $a=0.4$. We restrict ourselves to the central rapidity region, $|\eta | \le 2.5$, for the estimation of the $p_T$ flow density $\rho$. The SoftKiller subtraction is then applied to particles at the end of the parton shower, before jet clustering.

In addition, jet trimming [106], as implemented in FastJet, is applied to large-*R* jets. The trimming parameters are chosen such that the constituents of a given jet are reclustered into $k_T$ subjets with $R_{\text {sub}} = 0.2$. Subjets with transverse momentum less than 5 % of the total transverse momentum of the large-*R* jet are then removed. The use of trimming in addition to PU removal with SoftKiller is necessary to correct the jet mass in the boosted category, which is particularly susceptible to soft, wide-angle contaminations. No trimming is applied to the small-*R* jets and to the case without PU.

**Fig. 9:**
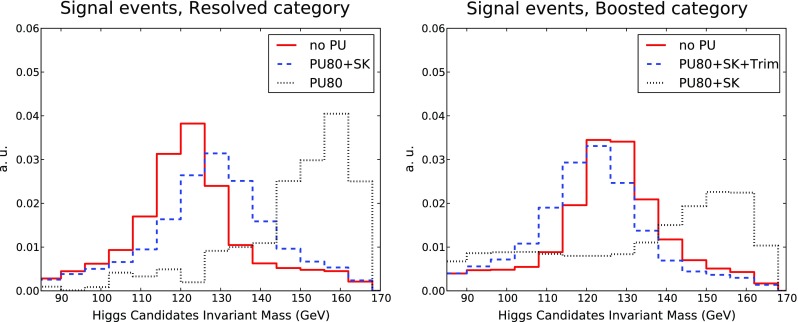
The invariant mass distributions of Higgs candidates in signal events in the resolved (*left*) and boosted (*right*) categories. In the resolved category, we compare the results without PU with those with PU80 with and without SK subtraction. In the boosted case, the comparison is performed between no PU, PU with only SK subtraction, and PU with both SK and trimming

**Fig. 10:**
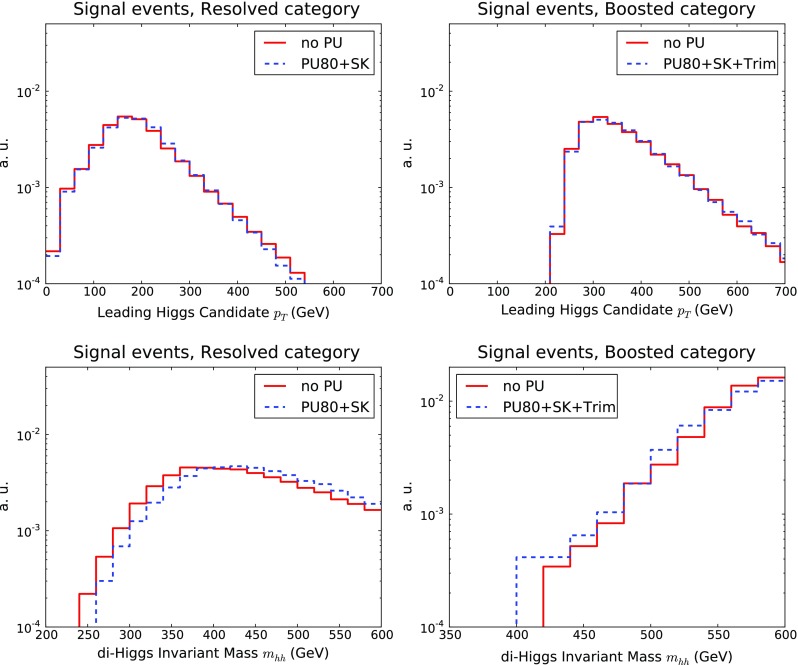
The transverse momentum $p_T^h$ of the leading Higgs candidate (*upper plots*) and of the invariant mass $m_{hh}$ of the di-Higgs system (*lower plots*) in the resolved (*left*) and boosted (*right*) categories. We compare the results without PU with those with PU80 and SK+Trim subtraction, as explained in the text

**Fig. 11:**
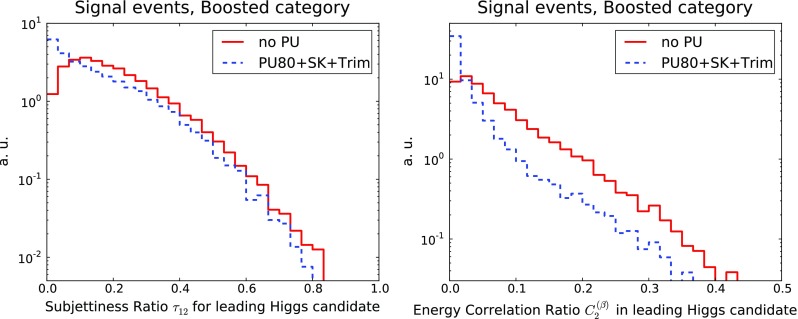
Same as Fig. 10 for the substructure variables $\tau _{21}$ (*left*) and $C_2^{(\beta )}$ (*right*) for the leading Higgs candidates in the boosted category

In Fig. 9 we show the invariant mass distributions of the Higgs candidates for signal events in the resolved and boosted categories. In the resolved category, we compare the results without PU with those with PU80, with and without SK subtraction. If PU is not subtracted, there is a large shift in the Higgs mass peak, by more than 30 GeV. Once SK subtraction is performed, we recover a distribution much closer to the no PU case, with only a small shift of a few GeV and a broadening of the mass distribution. In the boosted case, the comparison is performed between no PU, PU with only SK subtraction, and PU with both SK and trimming. We find that the mass distribution for jets to which no trimming is applied peaks at around 160 GeV, even after PU subtraction with SoftKiller. When trimming is applied in addition to SoftKiller, the distribution peaks close to the nominal Higgs mass, as in the case of the resolved category.

In Fig. 10 we compare the transverse momentum of the leading Higgs candidate, $p_T^{h}$ and the invariant mass of the di-Higgs system $m_{hh}$, in both the boosted and the resolved categories, between the no PU and the PU+SK+Trim cases. In the case of the $p_T^{h}$ distribution, the differences between the selection criteria for the resolved and boosted categories is reflected in the rightward shift of the latter. After subtraction, the effects of PU are small in the two categories. A similar behaviour is observed in the di-Higgs invariant mass distribution.

We can also assess the impact of PU on the substructure variables that will be used as input to the MVA in the boosted and intermediate categories. In Fig. 11 we show the 2-to-1 subjettiness ratio $\tau _{21}$, Eq. (5), and the ratio of energy correlation functions $C_2^{(\beta )}$, Eq. (6), for the leading Higgs candidate. We observe that the shapes of both substructure variables are reasonably robust in an environment including significant PU. Therefore we can consider the PU subtraction strategy as validated for the purposes of this study, although further optimisation should still be possible, both in terms of the SoftKiller and of the trimming input settings.

**Fig. 12:**
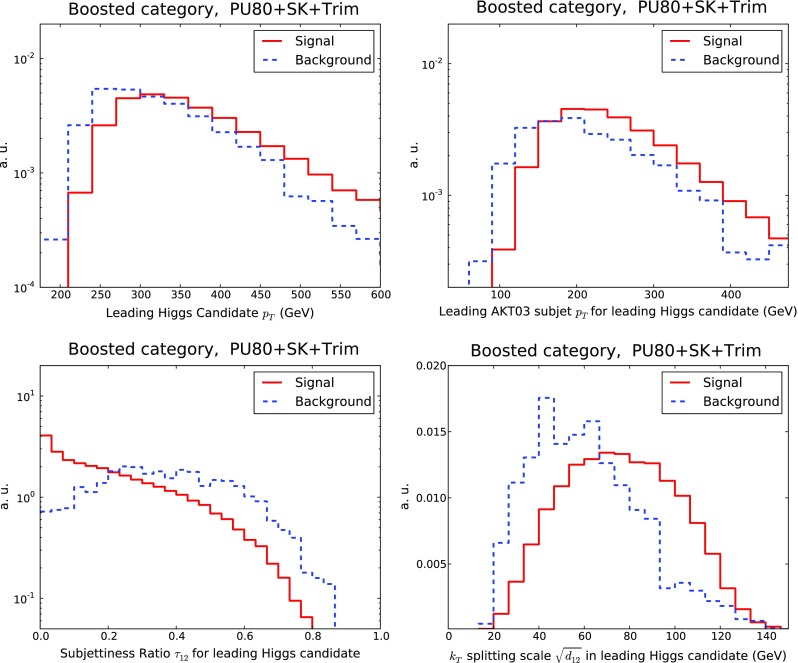
Comparison of kinematic distributions for the leading Higgs candidate, in the boosted category, for signal and background events in the case of PU subtraction with SK+Trim: its transverse momentum $p_T$, the $p_T$ of its leading AKT03 subjet, and the substructure variables $\tau _{21}$ and $\sqrt{d_{12}}$

It is also interesting to quantify how the relative differences between signal over background distributions are modified by the inclusion of PU. Considering the boosted category initially, in Fig. 12 we compare various kinematic distributions for signal and background events, with and without PU for the leading Higgs candidate: the transverse-momentum distribution $p_T$, the $p_T$ of the leading AKT03 subjet, the 2-to-1 subjettiness ratio $\tau _{21}$, and the $k_T$ splitting scale $\sqrt{d_{12}}$. We verify that the relevant qualitative differences between signal and background distributions are maintained in the presence of PU. This is especially noticeable for the substructure variables, which exhibit a similar discriminatory power both with and without PU.

**Fig. 13:**
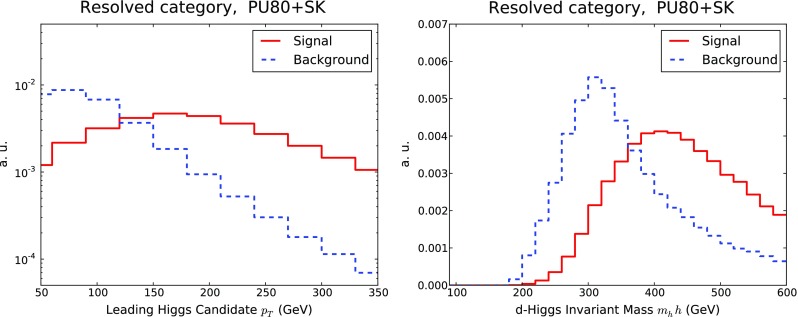
Same as Fig. 12 for the resolved category

We can also perform a similar comparison for the resolved category. In Fig. 13 we compare the kinematic distributions for signal and background events, with and without PU, for the invariant mass and the transverse momentum of the leading Higgs candidate. Again, the PU-subtracted background distributions appear reasonably close to their counterparts without PU, and thus the distinctive features between signal and background are maintained after PU subtraction.

It is illustrative to determine the mass resolution obtained for the reconstructed Higgs candidates in the various cases considered in the present study. In Table 2 we indicate the shift of the fitted invariant mass peak as compared to the nominal Higgs mass, $\langle m_h^\mathrm{reco}\rangle -m_h$, and the corresponding width of the distribution, $\sigma _{m_h}$, obtained from fitting a Gaussian to the mass distributions of leading and subleading Higgs candidates in the resolved and boosted categories. We show results for three cases: without PU, with PU80 but without subtraction (only for the resolved category), and the same with SK+Trim subtraction.

In both categories, we find a mass resolution of around 9 GeV in the case without PU. In the case of PU with SK+Trim subtraction, in the resolved category the mass resolution worsens only slightly to around 11 GeV, while in the boosted category we find the same resolution as in the no PU case. We also note that after SK+Trim subtraction, the peak of the invariant mass distributions of Higgs candidates coincides with the nominal values of $m_h$ within a few GeV for the two categories.

## Pre-MVA loose cut-based analysis

In this section we present the results of the pre-MVA loose cut-based analysis described in the previous section, and provide cut flows for the different analysis steps. We study how the signal significance is affected if only the 4*b* component of the QCD multi-jet background is taken into account. This section presents the results in an environment without pileup; the following one contains those obtained including significant PU.

**Table 2:** Resolution of the invariant mass distribution of reconstructed Higgs candidates in the resolved and boosted categories. We show three cases: no PU, with PU80 without subtraction (only for resolved), and the same with SK+Trim subtraction. We indicate the shift of the fitted invariant mass peak $\left\langle m_h^\mathrm{reco}\right\rangle$ for the Higgs candidates as compared to the nominal Higgs mass $m_h$, as well as the fitted Gaussian width $\sigma _{m_h}$

	$\left\langle m_h^\mathrm{reco}\right\rangle -m_h$ (GeV)	$\sigma _{m_h}$ (GeV)
*Resolved category*		
No PU		
Leading *h*	−3.8	$\left( 8.5\pm 0.2\right)$
Subleading *h*	−5.8	$\left( 9.1\pm 0.3\right)$
PU80		
Leading *h*	$+$33	$\left( 8.8\pm 1.5\right)$
Subleading *h*	$+$31	$\left( 11.7\pm 3.3\right)$
PU80+SK		
Leading *h*	$+$3.9	$\left( 10.7\pm 0.3\right)$
Subleading *h*	$+$2.1	$\left( 10.5\pm 0.3\right)$
*Boosted category*		
No PU		
Leading *h*	$+$2.0	$\left( 8.2\pm 0.5\right)$
Subleading *h*	$+$1.0	$\left( 8.8\pm 0.5\right)$
PU80+SK+Trim		
Leading *h*	−2.2	$\left( 8.7\pm 0.7\right)$
Subleading *h*	−4.9	$\left( 9.0\pm 0.8 \right)$

**Table 3:** Definition of the cuts imposed successively for the three selections

	Boosted	Intermediate	Resolved
**C1a**	$N_\mathrm{jets}^{R10}\ge 2$	$N_\mathrm{jets}^{R04}\ge 2$, $N_\mathrm{jets}^{R10}=1$	$N_\mathrm{jets}^{R04}\ge 4$
		+$p_T$ cuts and rapidity cuts	
**C1b**	+$N_\mathrm{MDT}\ge 2$	+$N_\mathrm{jets}^{R10}=1$ with MDT	+Higgs reconstruction
		+Higgs reconstruction	
**C1c**	+$m_h$ window cut		
**C2**	+*b*-tagging		

### Cut flow and signal significance

Here we compare the cross sections for signal and background events at various stages of the analysis. We consider all relevant backgrounds (see Sect. 2), and discuss how results are modified in the case where only the 4*b* background is considered. In Table 3 the different steps of the cut flow in the present analysis are summarised, separated into the boosted, intermediate, and resolved topologies. The different analysis steps proceed as follows:

- **C1a**: check that we have at least two large-*R* jets (in the boosted case), one large-*R* jet and at least 2 small-*R* jets (in the intermediate case) and at least four small-*R* jets (in the resolved case). In addition, require that these jets satisfy the corresponding $p_T$ thresholds; $p_T \ge 200$ GeV for large-*R* jets and $p_T \ge 40$ GeV for small-*R* jets, as well as the associated rapidity acceptance constraints.
- **C1b**: the two leading large-*R* jets must be mass-drop tagged in the boosted category. In the intermediate category, the large-*R* jet must also be mass-drop tagged.
- **C1c**: after the two Higgs candidates have been reconstructed, their invariant masses are required to lie within a window around $m_H$, in particular between 85 and 165 GeV, Eq. (8).
- **C2**: the *b*-tagging conditions are imposed (see Sect. 3.2), and the event is categorised exclusively into one of the three topologies, according to the hierarchy determined in Sect. 3.3.

Signal and background events satisfying all the analysis cuts up to the C2 level are then used as input for the MVA training, to be described next in Sect. 5.

In Table 4 we collect the values for the signal and background cross sections at the different analysis steps. Results are divided into the resolved, intermediate and boosted categories, and they are inclusive up to the C2 level, where exclusivity is imposed. In Table 4 we also provide the signal over background ratio, *S* / *B*, and the signal significance, $S/\sqrt{B}$, corresponding to an integrated luminosity of ${\mathcal {L}}=3$ ab$^{-1}$. These are computed either taking into account all the background components or the 4*b* QCD background only. We find that after *b*-tagging, the 2*b*2*j* component is of the same order of magnitude as the 4*b* component in all categories. This implies that the signal significance at the end of the cut-based analysis is degraded due to the contribution of light and charm jets being mis-identified as *b*-jets.

**Table 4:** The cross sections for the signal and the background processes at different steps of the analysis (see Table 3), for the resolved (upper), intermediate (middle) and boosted (lower table) categories, for the analysis without PU. For each step, the signal over background ratio *S* / *B*, and the signal significance $S/\sqrt{B}$ for ${\mathcal {L}}=3$ ab$^{-1}$ are also provided, considering either the total background, or only the 4*b* component

	*hh*4*b*	Total bkg	Cross section [fb]				*S* / *B*		$S/\sqrt{B}$	
			4*b*	2*b*2*j*	4*j*	$t\bar{t}$	Tot	4*b*	Tot	4*b*
*HL-LHC*, *resolved category*, *no PU*										
C1a	9	$2.2\times 10^8$	$6.9\times 10^4$	$1.5\times 10^7$	$2.0\times 10^8$	$2.1\times 10^5$	$4.0\times 10^{-8}$	$1.3\times 10^{-4}$	0.03	1.9
C1b	9	$2.2\times 10^8$	$6.9\times 10^4$	$1.5\times 10^7$	$2.0\times 10^8$	$2.1\times 10^5$	$4.0\times 10^{-8}$	$1.3\times 10^{-4}$	0.03	1.9
C1c	2.6	$4.4\times 10^7$	$1.6\times 10^4$	$3.2\times 10^6$	$4.1\times 10^7$	$8.8\times 10^4$	$6.1\times 10^{-8}$	$1.6\times 10^{-4}$	0.02	1.1
C2	0.5	$4.9\times 10^3$	$1.7\times 10^3$	$2.9\times 10^3$	$2.1\times 10^2$	47	$1.1\times 10^{-4}$	$2.9\times 10^{-4}$	0.4	0.6
*HL-LHC*, *intermediate category*, *no PU*										
C1a	2.8	$8.4\times 10^7$	$2.1\times 10^4$	$5.3\times 10^6$	$7.9\times 10^7$	$3.3\times 10^4$	$3.4\times 10^{-8}$	$1.3\times 10^{-4}$	0.02	1.1
C1b	2.6	$5.8\times 10^7$	$1.4\times 10^4$	$3.6\times 10^6$	$5.5\times 10^7$	$3.0\times 10^4$	$4.5\times 10^{-8}$	$1.9\times 10^{-4}$	0.02	1.2
C1c	0.5	$3.5\times 10^6$	$8.7\times 10^2$	$2.1\times 10^5$	$4.3\times 10^7$	$8.8\times 10^3$	$1.6\times 10^{-7}$	$6.1\times 10^{-4}$	0.02	1.0
C2	0.09	$1.8\times 10^2$	56	96	22	3.1	$5.3\times 10^{-4}$	$1.6\times 10^{-3}$	0.4	0.6
*HL-LHC*, *boosted category*, *no PU*										
C1a	3.9	$4.6\times 10^7$	$1.1\times 10^4$	$2.9\times 10^6$	$4.3\times 10^7$	$2.4\times 10^4$	$8.2\times 10^{-8}$	$3.4\times 10^{-4}$	0.03	2.0
C1b	2.7	$3.7\times 10^7$	$7.5\times 10^3$	$2.1\times 10^6$	$3.5\times 10^7$	$2.2\times 10^4$	$7.4\times 10^{-8}$	$3.7\times 10^{-4}$	0.03	1.7
C1c	1.0	$3.9\times 10^6$	$8.0\times 10^2$	$2.3\times 10^5$	$3.7\times 10^6$	$7.1\times 10^3$	$2.6\times 10^{-7}$	$1.3\times 10^{-3}$	0.03	2.0
C2	0.16	$2.5\times 10^2$	53	$1.9\times 10^2$	13	1.6	$5.7\times 10^{-4}$	$2.7\times 10^{-3}$	0.5	1.1

In the boosted category, at the end of the loose cut-based analysis, we find that around 500 events are expected at the HL-LHC, with a large number, ${\simeq } 10^6$, of background events. This leads to a pre-MVA signal significance of $S/\sqrt{B}=0.5$ and a signal over background ratio of $S/B=0.06~\%$. From Table 4 it is also possible to compute the corresponding pre-MVA expectations for the LHC Run II with ${\mathcal {L}}=300$ fb$^{-1}$: one expects in the boosted category around 50 signal events, with signal significance dropping down to $S/\sqrt{B}\simeq 0.16$. Such signal significances could have been enhanced by applying tighter selection requirements, but our analysis cuts have been left deliberately loose so that such optimisation may be performed by the MVA.

The resolved category benefits from higher signal yields, but this enhancement is compensated for by the corresponding increase in the QCD multi-jet background. In both resolved and intermediate categories the signal significance is $S/\sqrt{B}\simeq 0.4$, similar to that of the boosted category. A further drawback of the resolved case is that *S* / *B* is substantially reduced as compared to the boosted and intermediate cases.

Combining the results from the boosted, intermediate and resolved categories, we obtain an overall pre-MVA significance for the observation of the Higgs pair production in the $b\bar{b}b\bar{b}$ final state at the HL-LHC of $(S/\sqrt{B})_\mathrm{tot} \simeq 0.8$.

### The role of light and charm jet mis-identification

One of the main differences in the present study as compared to previous work is the inclusion of both irreducible and reducible background components, which allows us to quantify the impact of light and charm jet mis-identification. Two recent studies that have also studied the feasibility of SM Higgs pair production in the $b\bar{b}b\bar{b}$ final state are from the UCL group [28] and from the Durham group [29]. The UCL study is based on requiring at least four *b*-tagged $R=0.4$ anti-$k_T$ jets in central acceptance with $p_T \ge 40$ GeV, which are then used to construct dijets (Higgs candidates) with $p_T \ge 150$ GeV, $85 \le m_\mathrm{dijet} \le 140$ GeV and $\Delta R \le 1.5$ between the two components of the dijet. In addition to the basic selection cuts, the constraints from additional kinematic variables are included by means of a Boosted Decision Tree (BDT) discriminant. The backgrounds included are the 4*b* and 2*b*2*c* QCD multijets, as well as $t\bar{t}$, *Zh*, $t\bar{t}h$ and $hb\bar{b}$. For the HL-LHC, a signal significance of $S/\sqrt{B}\simeq 2.1$ is obtained.

The Durham group study [29] requires events to have two $R=1.2$ C/A jets with $p_T\ge 200$ GeV, and in addition two *b*-tagged subjets inside each large-*R* jet with $p_T \ge 40$ GeV each. To improve the separation between signal and background, both the BDRS method and the Shower Deconstruction (SD) [116, 117] technique are used. The backgrounds considered are QCD 4*b* as well as $Zb\bar{b}$, *hZ* and *hW*. At the HL-LHC, their best result is obtained by requiring two SD-tagged large-*R* jets, which leads to $S/\sqrt{B}\simeq 2.1$. Using the BDRS tagger results in slightly poorer performance.

**Fig. 14:**
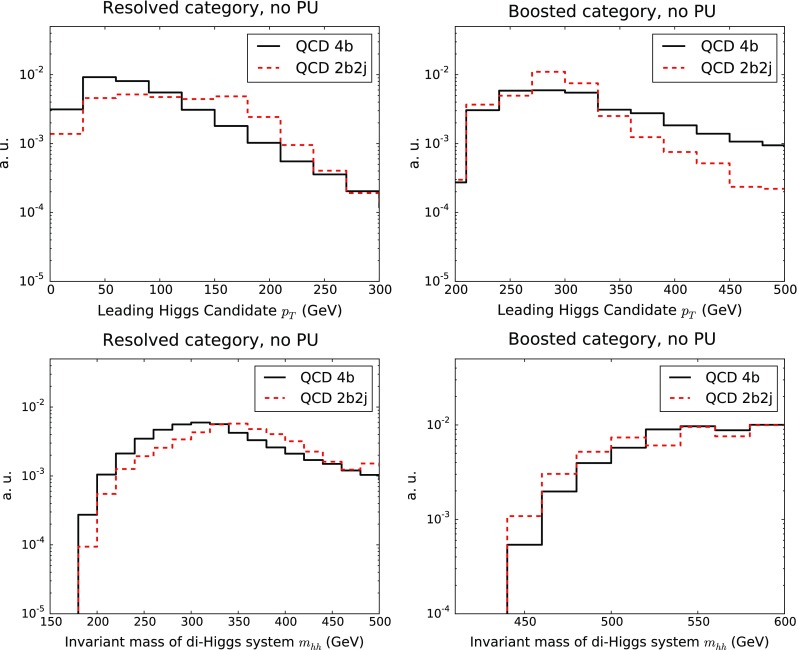
*Upper plots* Comparison of the shapes of the 4*b* and 2*b*2*j* components of the QCD background for the $p_T^h$ of the leading Higgs candidate in the resolved (*left plot*) and boosted (*right plot*) categories. *Lower plots* Same comparison for the invariant mass $m_{hh}$ of the reconstructed di-Higgs system

From our results in Table 4, we observe that the signal significance for the boosted, intermediate, and resolved categories is increased to 1.1, 0.6 and 0.6, respectively, when only the QCD 4*b* background is included. Combining the signal significance in the three categories, we obtain $(S/\sqrt{B_\mathrm{4b}})_\mathrm{tot}\simeq 1.4$, twice as large as the result found when all background components are included. Note the importance of the combination of the three exclusive event topologies, as opposed the exploitation of a single specific category. Taking into account the loose selection cuts, we see that our pre-MVA results including only the 4*b* background are consistent with those reported in previous studies.

From Table 4 we can compare the interplay between the reducible and irreducible components of the QCD backgrounds. In all cases, the 4*b* and 2*b*2*j* components have comparable magnitudes within the uncertainties from missing higher-order corrections. On the other hand, the 4*j* component is always substantially smaller. So while the 4*j* component can be safely neglected, the inclusion of the 2*b*2*j* component is essential to assess the feasibility of measuring Higgs pairs in this final state robustly, especially in the boosted category. This has the important consequence that a promising avenue to improve the prospects of this measurement would be to reduce, as much as possible, the light and charm jet mis-identification rate.

**Table 5:** The relative fractions $n^{(\text {b-jet})}_j$ of events for the resolved selection for which out of the four leading small-*R* jets of the event, *j* jets contain at least one *b*-quark with $p_T^b\ge 15$ GeV. This information is provided for the di-Higgs signal events and for the three QCD background samples. The last column indicates the overall selection efficiency as defined in Eq. (10)

	$n^{(\text {b-jet})}_0$ (%)	$n^{(\text {b-jet})}_1$ (%)	$n^{(\text {b-jet})}_2$ (%)	$n^{(\text { b-jet})}_3$ (%)	$n^{(\text {b-jet})}_4$ (%)	$\mathrm{EFF}_{\text {b-tag}}$ (%)
$hh\rightarrow 4b$	0.1	3	25	53	20	8.5
QCD 4*b*	1	8	27	44	20	8.4
QCD 2*b*2*j*	9	42	49	1	0.1	0.04
QCD 4*j*	96	3.5	0.5	0.01	$3\times 10^{-4}$	$2\times 10^{-4}$

In Fig. 14 we show a comparison of the shapes of the 4*b* and 2*b*2*j* components of the QCD background for the transverse momentum $p_T^h$ of the leading Higgs candidate and for invariant mass $m_{hh}$ of the reconstructed di-Higgs system in the resolved and boosted categories. The two components possess a rather similar shape for the two distributions, albeit with some differences. In the boosted category, the 4*b* component exhibits a less steep fall-off of the $p_T^h$ distribution at large $p_T$, while in the resolved case the 2*b*2*j* component has a slightly harder distribution of the invariant mass $m_{hh}$. We also observe that the 2*b*2*j* distributions are affected by somewhat larger Monte Carlo fluctuations as compared to 4*b*, despite the large size of the initial sample.

In the resolved category, the cross section before *b*-tagging is two orders of magnitude larger in the 2*b*2*j* sample as compared to the 4*b* sample. After *b*-tagging, a naive assessment would suggest a suppression of the 2*b*2*j* cross section by a factor $(f_l/f_b)^2 \simeq 1.5\times 10^{-4}$, as compared to the 4*b* component, since a total of four *b*-tags are required to classify the event as a Higgs candidate. In this case the ratio of 2*b*2*j* over 4*b* would be around ${\simeq } 3~\%$, and therefore negligible. While we have checked that this expectation is borne out at the parton level, we find that when parton shower effects are accounted for the situation is different, due both to radiation of $b\bar{b}$ pairs and from selection effects. Due to these, the number of *b* quarks in the final state is increased substantially in the 2*b*2*j* component as compared to the parton level, while at the same time the number of events in the 4*b* sample with 4 *b*-jets passing selection cuts is reduced.

We can make these statements more quantitative in the following way. To first approximation, neglecting the contribution from charm mis-identification, the overall efficiency of the *b*-tagging requirements in the resolved category will be given by the following expression:

10 $$\begin{aligned} \mathrm{EFF}_{\text {b-tag}}\simeq \sum _{j=0}^{4}n^{(\text {b-jet})}_j\cdot f_b^{j}\cdot f_l^{4-j}, \end{aligned}$$

with $n^{(\text {b-jet})}_j$ being the fraction of events satisfying all the selection requirements, where *j* jets out of the leading four jets of the event contain *b* quarks (with $p_T^b\ge 15$ GeV). Similar expressions can be derived for the boosted and intermediate categories.

The naive expectation is that all events in the 4*b* sample have $n^{(\text {b-jet})}_4\simeq 1$ and $n^{(\text {b-jet})}_j\simeq 0$ for $j\ne 4$, while the events in the 2*b*2*j* sample should have $n^{(\text {b-jet})}_2\simeq 1$ and zero otherwise. This leads to the ratio of overall *b*-tagging selection efficiencies

11 $$\begin{aligned} \frac{ \mathrm{EFF}_{\text {b-tag}} \left[ 2b2j \right] }{\mathrm{EFF}_{\text {b-tag}} \left[ 4b\right] } \simeq \left( \frac{f_l}{f_b}\right) ^2 \simeq 1.5\times 10^{-4}. \end{aligned}$$

However, after the parton shower, the above estimate is no longer accurate. First of all, we will have a non-negligible fraction $n^{(\text {b-jet})}_j$ with $j=3,4$ also in the 2*b*2*j* sample, due to *b*-quark pair radiation during the shower. Second, not all events in the 4*b* sample will lead to four small-*R* *b*-jets, due to a combination of selection cuts and parton shower effects.

In Table 5 we collect the values of $n^{(\text {b-jet})}_j$ for the signal and the three QCD background samples. We find that rather than the estimate Eq. (11), the correct ratio of *b*-tagging selection efficiencies is instead

12 $$\begin{aligned} \frac{\mathrm{EFF}_{\text {b-tag}} \left[ 2b2j\right] }{\mathrm{EFF}_{\text {b-tag}} \left[ 4b\right] }= \frac{0.04~\%}{8.4~\%} \simeq 5\times 10^{-3}. \end{aligned}$$

This suppression factor is of the same order as the ratio of 4*b* to 2*b*2*j* cross sections in the resolved category before *b*-tagging. This explains why the 2*b*2*j* contribution cannot be neglected as compared to the irreducible 4*b* component of the QCD background. A similar calculation from the numbers in Table 5 shows that, on the other hand, the 4*j* component of the background can be neglected.

## Multivariate analysis

At the end of the loose cut-based analysis, by combining the three event topologies, we obtain a signal significance of $S/\sqrt{B}\simeq 0.8~(1.4)$ with all backgrounds (only QCD 4*b*) considered. This section describes how this signal significance can be enhanced when the cut-based analysis is complemented by multivariate techniques. These are by now a mature tool in high-energy physics data analysis, opening new avenues to improve the performance of many measurements and searches at high-energy colliders. In particular, the classification of events into signal and background processes by means of MVAs is commonly used in LHC applications [28, 46, 80, 118–120].

In this section, first we present the specific MVA that we use, based on feed-forward multi-layer neural networks. Then we introduce the input variables that are used in the MVA, including the jet substructure variables, and then present the signal significance obtained by applying the MVA. Then we assess the robustness of the MVA strategy in the case of significant contamination from pileup.

### Deep artificial neural networks

The specific type of MVA that we use to disentangle signal and background events is a multi-layer feed-forward artificial neural network (ANN), known as a *perceptron*.^2^ This family of ANNs are also known as *deep neural networks*, due to their multi-layered architecture. The MVA inputs are a set of kinematic variables describing the signal and background events which satisfy the requirements of the cut-based analysis. The output of the trained ANNs also allows for the identification, in a fully automated way, of the most relevant variables in the discrimination between signal and background.

**Fig. 15:**
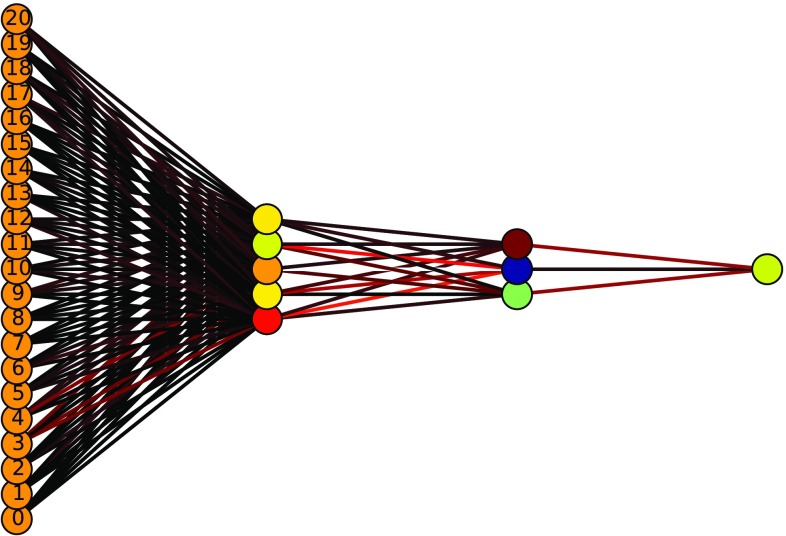
Schematic of the Artificial Neural Network (ANN) used for the analysis of the boosted category, with $N_\mathrm{var}=21$ input variables and thus the same number of neurons in the first layer. The *color code* in the neuron connections (the weights) is a heat map obtained at the end of the Genetic Algorithms training, with *red* indicating larger values and *black* indicating smaller values

In this work, the ANN that we use has the following architecture.

13 $$\begin{aligned} N_{\mathrm {var}}\times 5\times 3\times 1, \end{aligned}$$

where $N_{\mathrm {var}}$ represents the number of input variables for the MVA, which is different in the resolved, intermediate, and boosted categories. All neural-network layers use a sigmoid activation function, allowing for a probabilistic interpretation of the ANN output. In Fig. 15 we show an illustrative example of an ANN used in this work, corresponding to the case of the boosted category (thus $N_{\mathrm {var}}=21$, as we explain below).

The training of the ANN for the signal/background classification task proceeds as follows. Given a set of $N_{\mathrm {var}}$ kinematic variables $\{k\}_i$ associated with the event *i* and a set of neural-network weight parameters $\{\omega \}$, we interpret the neural-network output $y_i$ (the activation state of the neuron in the last layer) as the probability that the event *i* originates from the signal process,

14 $$\begin{aligned} y_i = P(y^\prime _i=1|\{k\}_i, \{\omega \} )\, , \end{aligned}$$

where $y_i^\prime$ represents the true classification of the event *i*, i.e., $y^\prime _i = 1$ for signal and $y^\prime _i = 0$ for background events. With this interpretation, our general classification probability including background events is given by

15 $$\begin{aligned} P(y_i^\prime |\{k\}_i, \{\omega \}) = y_i^{y^\prime _i}(1-y_i)^{1-y^\prime _i}, \end{aligned}$$

consequently we can define an error function $E(\{\omega \})$ to be minimised during the ANN training. In this case, the error function is the cross-entropy function, defined as

16 $$\begin{aligned} E(\{\omega \})\equiv & {} -\log \left( \prod _i^{N_{\text {ev}}} P(y_i^\prime |\{k\}_i, \{\omega \})\right) \nonumber \\= & {} \sum _i^{N_{\text {ev}}} \left[ y^\prime _i\log {y_i} + (1-y^\prime _i)\log {(1-y_i)}\right] , \end{aligned}$$

where $N_{\text {ev}}$ is the number of Monte Carlo events that are used for the ANN training. The ANN is trained both on the signal and background MC events, so it is important to ensure that the input MC sample is large enough to avoid contamination from MC statistical fluctuations.

The training of the neural networks therefore consists of the minimisation of the cross-entropy error, Eq. (16), which in this work is achieved using a Genetic Algorithm (GA). GAs [125–128] are non-deterministic minimisation strategies suitable for the solution of complex optimisation problems, for instance when a very large number of quasi-equivalent minima are present. GAs are inspired on natural selection processes that emulate biological evolution. In our case, the GA training is performed for a very large number of generations, $N_\mathrm{gen}=5\times 10^{4}$, to avoid the risk of under-training. We have verified that if a much larger number of generations are used, the results are unchanged.

In addition, in order to avoid the possibility of over-fitting, we have used a cross-validation stopping criterion, in particular the same one as that used in the NNPDF3.0 analysis [66]. This cross-validation proceeds by dividing the input MC dataset into two disjoint sets, using one for training the ANN and the other for validation: the optimal stopping point is then given by the minimum of the error function Eq. (16) to the validation sub-sample. This indicates the point where the ANN begins to train upon statistical fluctuations in the input MC samples, rather than learning the underlying (smooth) physical distributions.

**Fig. 16:**
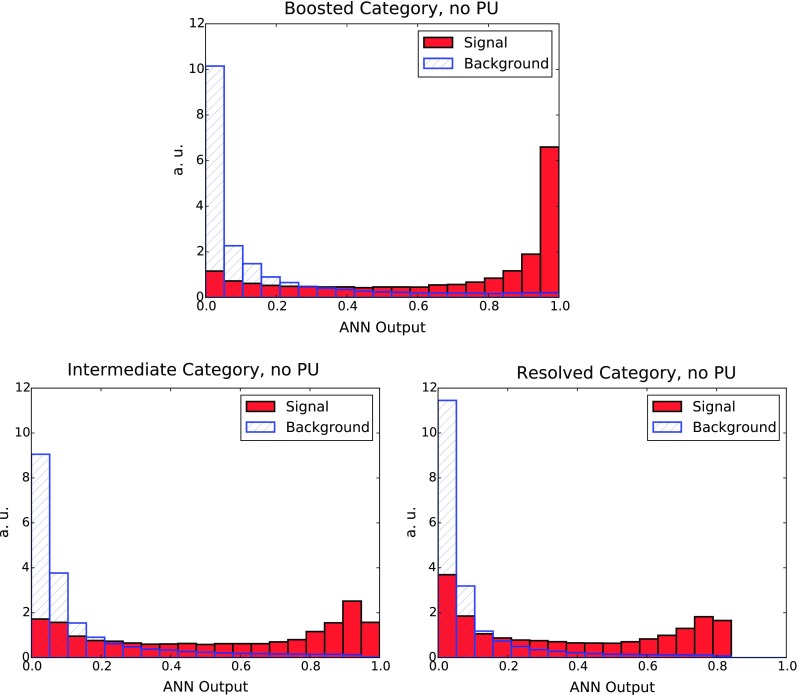
The distributions, at the end of the GA training, for the signal and background MC events in the three categories: boosted (*upper plot*), intermediate (*lower left plot*) and resolved (*lower right plot*), as a function of the ANN output

### Input kinematic variables

In this work we use different sets of input variables for the three categories. In the case of large-*R* jets, we exploit the available information on jet substructure. For the three categories, boosted, intermediate and resolved, the following common variables are used as input to the MVA:

- The transverse momenta of the leading and subleading Higgs, $p_{T,h_1}$ and $p_{T,h_2}$.
- The transverse momentum of the reconstructed Higgs pair, $p_{T,hh}$.
- The invariant masses of the leading and subleading Higgs candidates, $m_{h,1}$ and $m_{h,2}$.
- The invariant mass of the reconstructed Higgs pair, $m_{hh}$.
- The separation in the $\phi$–$\eta$ plane between the two Higgs candidates, $\Delta R_{hh}$.
- The separation in $\eta$ between the two Higgs candidates, $\Delta \eta _{hh}$.
- The separation in $\phi$ between the two Higgs candidates, $\Delta \phi _{hh}$.

In addition, in the boosted category we use the transverse momenta of the leading, $p_{T,h_{1,1}}$ and $p_{T,h_{1,2}}$ and subleading, $p_{T,h_{2,1}}$ and $p_{T,h_{2,2}}$, Higgs candidate AKT03 subjets. In the resolved category instead, the corresponding variables are the transverse momenta $p_{T,i}$ of the four leading *b*-tagged small-*R* jets in the event. In the intermediate category, we use the transverse momenta of the subjets from the large-*R* jet $p_{T,h_{1,1}}$ and $p_{T,h_{1,2}}$ and the transverse momenta $p_{T,i}$ of the two leading *b*-tagged small-*R* jets. Therefore, we have 13 variables which are common to the three categories.

**Fig. 17:**
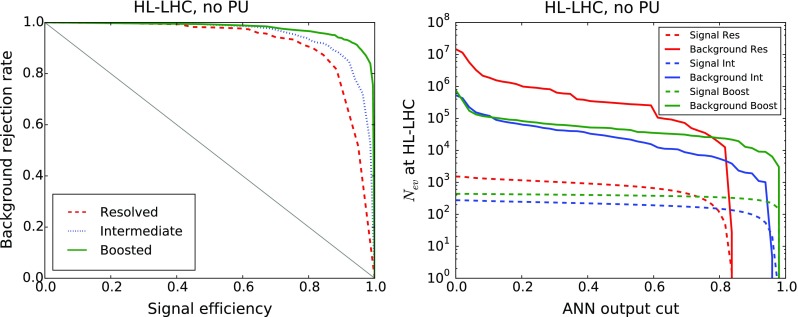
*Left* ROC curve for the background rejection rate as a function of the signal selection efficiency, as the cut $y_\mathrm{cut}$ in the ANN output is varied. *Right* Number of signal (*dashed*) and background (*solid*) events expected at the HL-LHC as a function of the $y_\mathrm{cut}$

In the boosted and intermediate categories, we also include the jet substructure variables introduced in Sect. 3 for the large-*R* jets: the $k_T$ splitting scales $\sqrt{d_{12}}$, the ratio of 2-to-1 subjettiness $\tau _{12}$, and the ratios of energy correlation functions $C^{(\beta )}_2$ and $D_2^{(\beta )}$. This leads to a total of $N_{\mathrm {var}}=13,17$ and 21 variables for the resolved, intermediate and boosted categories, respectively.

Given that the MVA is able to identify the most discriminatory variables in an automated way, and to suppress those which have little effect, it is advantageous to include a wide array of input variables. This is one of the main advantages of ANNs in this context: their inherent redundancy means that adding additional information, even if carries very little weight, should not degrade the classification power of the MVA.

### MVA results

We now present the results of the MVA, first without PU, and then later including the effects of PU. First of all, in Fig. 16 we show the distribution of the ANN output at the end of the GA minimisation, separately for the boosted, intermediate and resolved categories. All distributions are normalised so that their integral adds up to one. The separation between signal and background is achieved by introducing a cut, $y_\mathrm{cut}$, on the ANN output, so that MC events with $y_i\ge y_\mathrm{cut}$ are classified as signal events, and those with $y_i < y_\mathrm{cut}$ as background events. Therefore, the more differentiated the distribution of the ANN output is for signal and background events, the more efficient the MVA discrimination will be.

From Fig. 16 we see that in the boosted category the MVA can produce a clear discrimination between signal and background, with the two distributions forming peaks at their respective optimal limits. This indicates that introducing a suitable cut $y_\mathrm{cut}$ in the ANN output will substantially reduce the background, while keeping a reasonable signal efficiency. The performance of the MVA discrimination is similar, although slightly worse, in the intermediate and resolved categories.

The results for the signal selection efficiency and the background rejection rate as a function of the cut in the ANN output $y_\mathrm{cut}$ define the so-called Receiver-Operating Characteristic (ROC) curve, shown in Fig. 17. It is clear that we can achieve high signal efficiency by using a small value of $y_\mathrm{cut}$, but such a choice would be affected by poor background rejection. Conversely, using a higher value of the cut will increase background rejection at the cost of dropping signal efficiency. As could already be inferred from the distribution of neural-networks output in Fig. 16, we find that our MVA is reasonably efficient in discriminating signal over background. The performance is best in the case of the boosted category, and then slightly worse in the resolved and intermediate categories, consistent with the distributions of the ANN outputs in Fig. 16.

It is useful to estimate, for each value of the cut in the ANN output $y_\mathrm{cut}$, how many signal and background events are expected at the HL-LHC with ${\mathcal {L}}=3$ ab$^{-1}$. This comparison is shown in Fig. 17. We observe that in the boosted category, for a value $y_\mathrm{cut}\simeq 0.9$ we end up with around 300 signal events and $10^4$ background events. Similar results are obtained in the intermediate and resolved categories: in the former we find 130 ($3\times 10^3$) signal (background) events for $y_\mathrm{cut}\simeq 0.85$ (0.60), and in the latter 630 ($10^5$) signal (background) events for $y_\mathrm{cut}\simeq 0.6$. Therefore, the MVA achieves a substantial background suppression with only a moderate reduction of signal efficiency.

A useful property of MVAs such as the one used in our analysis is that they can provide direct physical insight about which of the input variables contribute to the separation between signal and background. In the case of ANNs, this can be quantified by computing the sum of the absolute values of all the weights connected to a given input neuron *i*, that is,

17 $$\begin{aligned} \omega ^\mathrm{(tot)}_i \equiv \sum _{k=1}^{n^{(2)}} \Big |\omega ^{(2)}_{ki}\Big |, \quad i=1,\ldots ,N_\mathrm{var}, \end{aligned}$$

with $\omega ^{(2)}_{ki}$ the value of the weight connecting the *k*th neutron of the second layer with the *i*th neuron of the first (input) layer, and $n^{(2)}=5$ the number of neurons in the second layer. Those input variables with a larger value of $\omega ^\mathrm{(tot)}_i$ will be those that play a more significant role in enhancing the signal discrimination using the MVA. We note, however, that the estimate provided by Eq. (17) is necessarily qualitative.

**Fig. 18:**
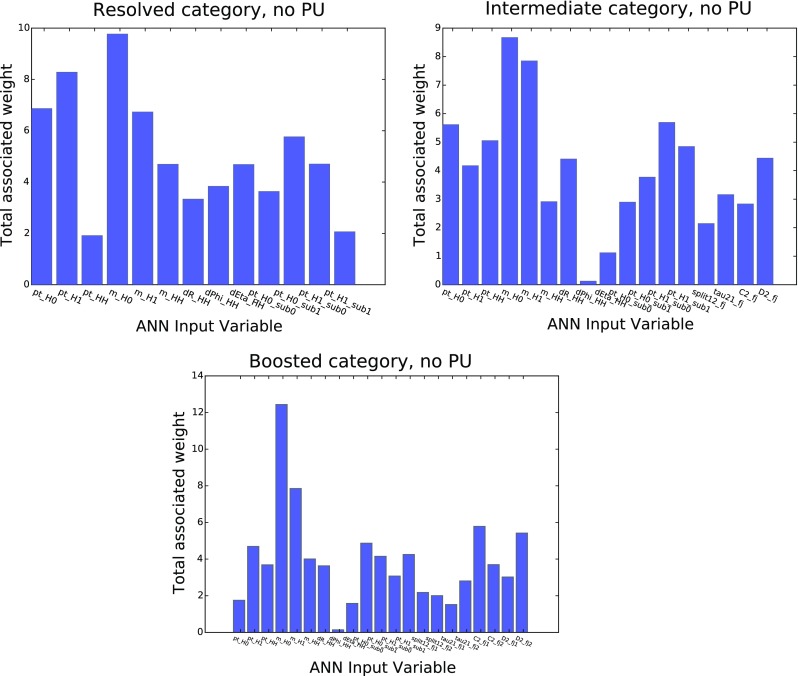
Distribution of the total associated weight, Eq. (17) for each of the $N_\mathrm{var}$ input variables of the resolved (*upper left*), intermediate (*upper right*) and boosted (*lower plot*) categories

In Fig. 18 we show the distribution of the total associated weight, Eq. (17) for each of the $N_\mathrm{var}$ input variables of the three categories, using the notation for the kinematic variables as in Sect. 5.2. In the resolved category, the variables that carry a higher discrimination power are the transverse momentum of the two reconstructed Higgs candidates and their invariant masses. In the case of the boosted category, the invariant mass distribution of the Higgs candidates is also the most discriminatory variable, followed by the subjet $p_T$ distributions and substructure variables such as $C_2^{(\beta )}$ and $D_2^{(\beta )}$.

**Fig. 19:**
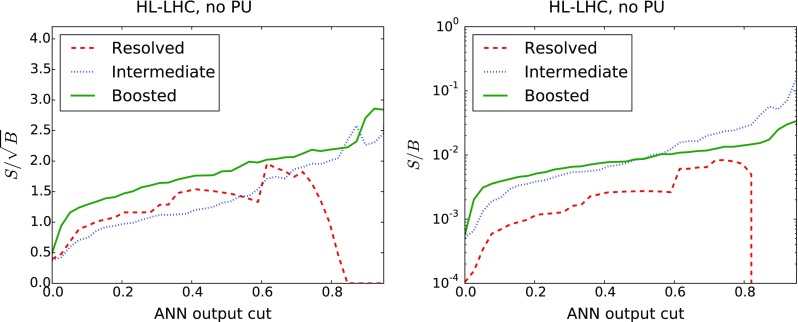
The values of the signal significance, $S/\sqrt{B}$, and of the signal over background ratio, *S* / *B*, for the boosted, intermediate and resolved categories as a function of the cut $y_\mathrm{cut}$ in the ANN output. The $y_\mathrm{cut}=0$ results are those at the end of the cut-based analysis

The results for the signal significance $S/\sqrt{B}$ and the signal over background ratio *S* / *B* as a function of $y_\mathrm{cut}$ for the three categories are given in Fig. 19. The values for $y_\mathrm{cut}=0$ correspond to those at the end of the loose cut-based analysis. We observe how in the three categories there is a marked improvement in signal significance as compared to the pre-MVA results. We also observe a substantial enhancement in *S* / *B*, arising from the background suppression achieved by the MVA, reaching values of 1, 6 and 3.5 % in the resolved, intermediate and boosted categories. This improvement in *S* / *B* is crucial to ensure the feasibility of this measurement, since it allows systematic uncertainties in the background determination to be at most of a similar size.

The optimal value of the cut in the ANN output, $y_\mathrm{cut}$, can be determined from the maximisation of $S/\sqrt{B}$, ensuring that the number of signal events $N_\mathrm{ev}$ expected at the HL-LHC does not become too low. In addition, we require that the number of MC events used to define the signal category (events with $y_i \ge y_\mathrm{cut}$) is sufficiently large in order to avoid the biases and statistical fluctuations associated to a small training sample. In Table 6 we quote, for the optimal value of $y_\mathrm{cut}$ in each category, the number of signal and background events $N_\mathrm{ev}$ expected at the HL-LHC, as well as $S/\sqrt{B}$ and *S* / *B*. For completeness, we also include the corresponding pre-MVA results.

**Table 6:** Post-MVA results, for the optimal value of the ANN discriminant $y_\mathrm{cut}$ in the three categories, compared with the corresponding pre-MVA results ($y_\mathrm{cut}=0$). We quote the number of signal and background events expected for ${\mathcal {L}}=3$ ab$^{-1}$, the signal significance $S/\sqrt{B}$ and the signal over background ratio *S* / *B*. The pre-MVA results correspond to row C2 in Table 4

HL-LHC, no PU				
Category	$N_\mathrm{ev}$ signal	$N_\mathrm{ev}$ back	$S/\sqrt{B}$	*S* / *B*
*Boosted*				
$y_\mathrm{cut}=0$	440	$7.6\times 10^5$	0.5	$6\times 10^{-4}$
$y_\mathrm{cut}=0.90$	290	$1.2\times 10^4$	2.7	0.03
*Intermediate*				
$y_\mathrm{cut}=0$	280	$5.3\times 10^5$	0.4	$5\times 10^{-4}$
$y_\mathrm{cut}=0.85$	130	$3.1\times 10^3$	2.3	0.04
*Resolved*				
$y_\mathrm{cut}=0$	1500	$1.5\times 10^{7}$	0.4	$1\times 10^{-4}$
$y_\mathrm{cut}=0.60$	630	$1.1\times 10^{5}$	1.9	0.01

From Table 6 we see that following the application of the MVA, the signal significance in the boosted category increases from 0.5 to 2.7, with *S* / *B* increasing from 0.06 to $3~\%$. For the intermediate and resolved categories, $S/\sqrt{B}$ increases from 0.4 to 2.3 and 1.9, respectively, with the signal over background ratio raising from 0.05 and $0.01~\%$ to 4 and 1 %. Combining the three categories, taking into account all background components, we obtain the overall signal significance:

18 $$\begin{aligned} \left( \frac{S}{\sqrt{B}}\right) _\mathrm{tot} \simeq 4.0~(1.3),\quad {\mathcal {L}}=3000~(300)\,\mathrm{fb}^{-1}. \end{aligned}$$

The signal significance for ${\mathcal {L}}=3$ ab$^{-1}$ is thus well above the threshold for the observation of Higgs pair production. However, given that the HL-LHC will be a high-PU environment, which will affect the description of the various kinematic distributions used as input to the MVA, it is essential to quantify the robustness of these results in a realistic environment including the effects of significant PU.

It should be emphasised that MVAs such as the ANNs used in this work can always be understood as a combined set of correlated cuts. Once the ANNs have been trained, it is possible to compare kinematical distributions after and before the ANN cut to verify its impact. This information would allow one in principle to perform a cut-based analysis, without the need of using ANNs, and finding similar results.

**Fig. 20:**
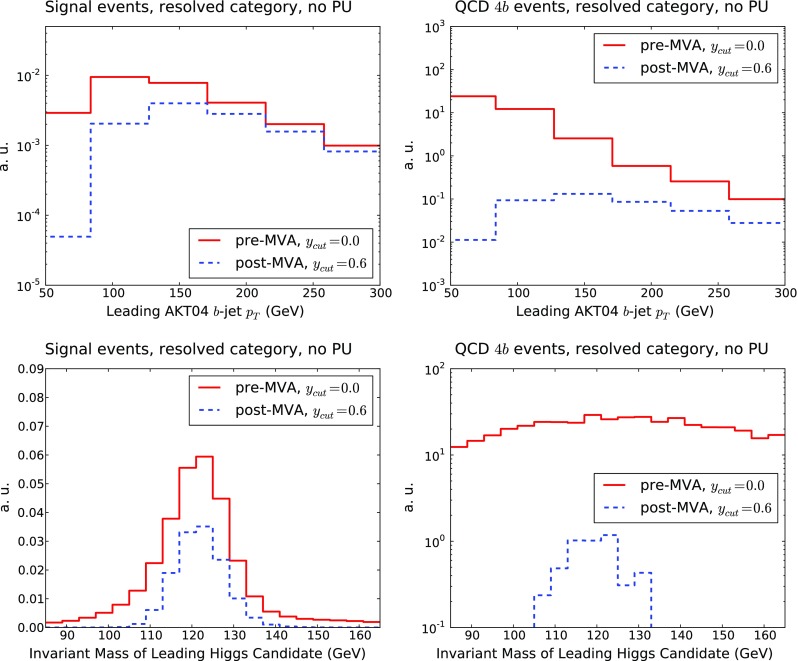
The $p_T$ distribution of the leading AKT04 small-*R* jets (*upper plots*) and the invariant mass of reconstructed Higgs candidates (*lower plots*) in the resolved category, comparing the pre-MVA results ($y_\mathrm{cut}=0$) with the post-MVA results ($y_\mathrm{cut}=0.60$) for signal (*left*) and background (*right plot*) events. In this case the distributions are not normalised, to better visualise the effects of the MVA cut

To illustrate this point, in Fig. 20 we show the $p_T$ distribution of the leading AKT04 small-*R* jets and the invariant mass of reconstructed Higgs candidates in the resolved category, comparing the pre-MVA results ($y_\mathrm{cut}=0$) with the post-MVA results ($y_\mathrm{cut}=0.60$) for signal and background events. The distributions are not normalised, to better visualise the effect of the MVA cut. Unsurprisingly, the ANN cut effectively selects events which lead to similar kinematical distributions between signal and background events. In the case of the small-*R* jets $p_T$ distribution, the ANN cuts favours the high-$p_T$ region, while for the invariant mass distribution only the region around the Higgs mass peak is selected for background events.

A particularly challenging aspect of our analysis is the modeling of the 2*b*2*j* and 4*j* background, especially for the latter, which require extremely large MC samples. In the analysis reported here, out of the original 3M 4*j* generated events, only around 100 survive the analysis cuts, and thus these low statistics have associated a potentially large uncertainty in the calculation of the post-MVA 4*j* cross section. On the other hand, since the 4*j* cross sections are always quite smaller than the sum of the 4*b* of the 2*b*2*j* components, these low statistics should not modify qualitatively our conclusions above. To verify explicitly this expectation, and obtain a more robust estimate of the background cross section from mis-identified jets, we have increased by a factor 10 the size of the 2*b*2*j* and 4*j* background samples, up to a total of 30M each. Processing these events though our analysis, including retraining the MVA, we find $(S/\sqrt{B})_\mathrm{tot}=3.9$, consistent with Eq. (18), indicating that the low statistics of the 4*j* background is not a limiting factor.

### Impact of PU in the MVA

In this section we study how the MVA results are modified when the analysis is performed including significant PU. The loose cut-based analysis and the subsequent MVA optimisation have been performed using the same settings as in the case without PU. In Table 7 we provide the pre-MVA cut flow in the case of PU80, the corresponding version without PU being Table 4. The interplay between the signal cross sections and the various background components is qualitatively unchanged as compared to the no PU case.

**Table 7:** Same as Table 4, now for the case of PU80+SK+Trim

	*hh*4*b*	Total bkg	Cross section [fb]				*S* / *B*		$S/\sqrt{B}$	
			4*b*	2*b*2*j*	4*j*	$t\bar{t}$	Tot	4*b*	Tot	4*b*
*HL-LHC*, *resolved category*, *PU+SK with* $n_\mathrm{PU}=80$										
C1a	11	$4.4 \times 10^8$	$1.5\times 10^5$	$3.0\times 10^7$	$4.1\times 10^8$	$2.6 \cdot 10^5$	$2.4 \times 10^{-8}$	$7.2 \times 10^{-5}$	0.03	1.5
C1b	11	$4.4 \times 10^8$	$1.5\times 10^5$	$3.0\times 10^7$	$4.1\times 10^8$	$2.6 \cdot 10^5$	$2.4 \times 10^{-8}$	$7.2 \times 10^{-5}$	0.03	1.5
C1c	3	$1.1 \times 10^8$	$4.2\times 10^4$	$7.7\times 10^6$	$9.9\times 10^7$	$1.1 \cdot 10^5$	$2.8 \times 10^{-8}$	$7.4 \times 10^{-5}$	0.02	0.8
C2	0.6	$9.0 \times 10^3$	$3.5\times 10^3$	$5.1\times 10^3$	$3.1\times 10^2$	50	$6.5 \times 10^{-5}$	$1.7 \times 10^{-4}$	0.4	0.5
*HL-LHC*, *intermediate category*, *PU+SK+Trim with* $n_\mathrm{PU}=80$										
C1b	2.7	$8.1\times 10^7$	$2.1\times 10^4$	$5.2\times 10^6$	$7.6\times 10^7$	$3.0\times 10^4$	$3.4 \times 10^{-8}$	$1.3 \times 10^{-4}$	0.02	1.0
C1c	2.6	$6.2\times 10^7$	$1.5\times 10^4$	$3.9\times 10^6$	$5.8\times 10^7$	$2.8\times 10^4$	$4.1 \times 10^{-8}$	$1.7 \times 10^{-4}$	0.02	1.1
C1d	0.5	$2.8\times 10^6$	$7.9\times 10^2$	$1.9\times 10^5$	$2.7\times 10^6$	$6.5\times 10^3$	$1.8 \times 10^{-7}$	$6.2 \times 10^{-4}$	0.02	1.0
C2	0.09	$2.6\times 10^2$	47	$1.8\times 10^2$	30	2.2	$3.4 \times 10^{-4}$	$1.8 \times 10^{-3}$	0.3	0.7
*HL-LHC*, *boosted category*, *PU+SK+Trim with* $n_\mathrm{PU}=80$										
C1a	3.5	$4.1\times 10^7$	$1.0\times 10^4$	$2.7\times 10^6$	$3.8\times 10^7$	$2.0\times 10^4$	$8.6 \times 10^{-8}$	$3.4 \times 10^{-4}$	0.03	1.9
C1b	2.5	$3.2\times 10^7$	$6.8\times 10^3$	$1.9\times 10^6$	$3.0\times 10^7$	$1.9\times 10^4$	$7.8 \times 10^{-8}$	$3.6 \times 10^{-4}$	0.02	1.6
C1c	0.8	$2.2\times 10^6$	$5.4\times 10^2$	$1.4\times 10^5$	$2.0\times 10^6$	$4.8\times 10^3$	$3.8 \times 10^{-7}$	$1.6 \times 10^{-3}$	0.03	2.0
C2	0.14	$1.5\times 10^2$	40	86	22	1.8	$9.0 \times 10^{-4}$	$3.5 \times 10^{-3}$	0.6	1.2

**Table 8:** Same as Table 6, now for the case of PU80+SK+Trim

HL-LHC, PU80+SK+Trim				
Category	$N_\mathrm{ev}$ signal	$N_\mathrm{ev}$ back	$S/\sqrt{B}$	*S* / *B*
*Boosted*				
$$y_\mathrm{cut}=0$$	410	$$4.5\times 10^5$$	0.6	$$ 10^{-3}$$
$y_\mathrm{cut}=0.8$	290	$3.7\times 10^4$	1.5	0.01
*Intermediate*				
$y_\mathrm{cut}=0$	260	$7.7\times 10^5$	0.3	$3\times 10^{-4}$
$y_\mathrm{cut}=0.75$	140	$5.6\times 10^3$	1.9	0.03
*Resolved*				
$y_\mathrm{cut}=0$	1800	$2.7\times 10^7$	0.4	$7\times 10^{-5}$
$y_\mathrm{cut}=0.60$	640	$1.0\times 10^5$	2.0	0.01

In Table 8 we compare the results for the PU80+SK+Trim case between the pre-MVA loose cut-based analysis and the post-MVA results for the optimal values of the ANN output cut $y_\mathrm{cut}$. As in Table 6, we also quote the number of signal and total background events expected for ${\mathcal {L}}=3$ ab$^{-1}$ and the values of $S/\sqrt{B}$ and *S* / *B*. We observe that the pre-MVA signal significance is close to the results of the simulations without PU for the three categories. We now find values for $S/\sqrt{B}$ of 0.4, 0.3 and 0.6, in the resolved, intermediate and boosted categories, respectively, to be compared with the corresponding values without PU, namely 0.4, 0.4 and 0.5. The number of selected signal events in each category at the end of the cut-based analysis is only mildly affected by PU. The slight pre-MVA improvement in $S/\sqrt{B}$ for the boosted case arises from a reduction in the number of background events that are classified in this category as compared to the case without PU.

Once the MVA is applied, the signal significance in the resolved, intermediate and boosted categories increases to 2.0, 1.9 and 1.5 respectively, to be compared with the corresponding values without PU, namely 1.9, 2.3 and 2.7. Therefore, the post-MVA effect of PU on $S/\sqrt{B}$ is a moderate degradation of the boosted and intermediate categories, especially for the former, while the resolved category is largely unchanged.^3^ We also observe that, due to the MVA, the signal over background ratio is increased from 0.007, 0.03 and 0.1 % up to 1, 3 and 1 % in the resolved, intermediate and boosted categories, respectively. This indicates that, while this measurement is still highly challenging, requiring a careful extraction of the QCD background from the data, it should be within reach.

**Fig. 21:**
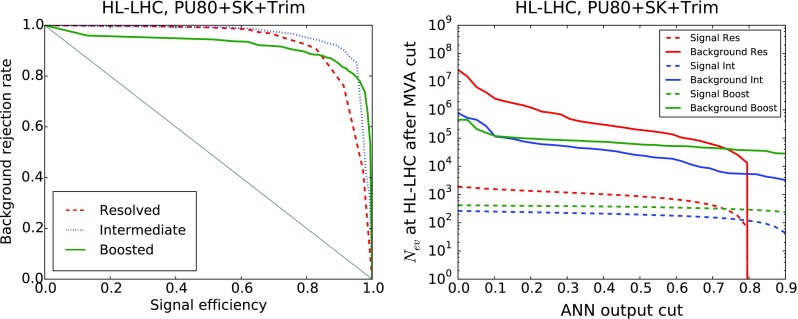
Same as Fig. 17 for the PU80+SK+Trim case

**Fig. 22:**
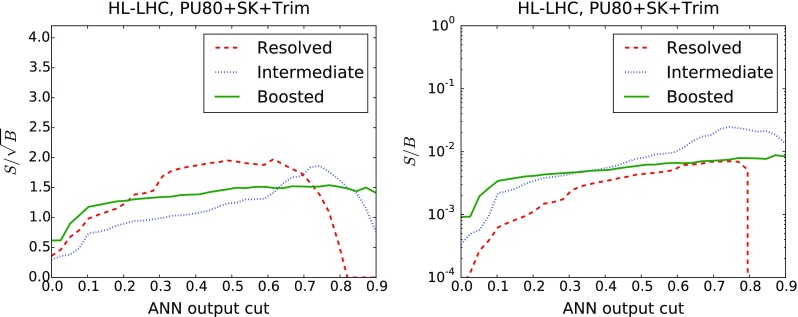
Same as Fig. 19 for the PU80+SK+Trim case

In Fig. 21 we show the number of signal and background events that are expected for ${\mathcal {L}}=3$ ab$^{-1}$ as a function of $y_\mathrm{cut}$, together with the corresponding ROC curve. The slight degradation of the boosted category in the case of PU can be seen by comparing with the corresponding results without PU in Fig. 17. In Fig. 22 we show the signal significance, $S/\sqrt{B}$, and the signal over background ratio, *S* / *B*, accounting now for the effects of PU. The corresponding results in the case without PU were shown in Fig. 19. As can be seen, the MVA-driven enhancement remains robust in the presence of PU, with $S/\sqrt{B}$ only moderately degraded. Therefore, the qualitative conclusions drawn in the case without PU also hold when the analysis is performed in a high-PU environment. Since no specific effort has been made to optimise PU subtraction, for instance by tuning the values of the patch length *a* in SoftKiller or the $p_T$ threshold during jet trimming, we believe that there should be still room for further improvement.

It is useful to quantify which of the MVA input variables carry the highest discrimination power in the case of PU, by means of Eq. (17), and compare this with the corresponding results without PU shown in Fig. 18. We have verified that the relative weight of the different input variables to the MVA is mostly unchanged in the case of PU. In the resolved category, the highest total associated weight is carried by the Higgs candidates $p_T$ and invariant mass, as well as by the $p_T$ of the individual small-*R* jets. For the boosted category, the highest weight is carried by the Higgs invariant mass, followed by the Higgs $p_T$,  $m_{hh}$, the $p_T$ of the AKT03 subjets and the substructure variables, with a similar weighting among them.

In Table 9 we provide the post-MVA number of signal and background events expected for ${\mathcal {L}}=3$ ab$^{-1}$. For the backgrounds, we quote both the total number, $N_\mathrm{ev}^\mathrm{tot}$, and the QCD 4*b* component only, $N_\mathrm{ev}^\mathrm{4b}$. We quote results for the no PU and PU80+SK+Trim cases. We also quote in each case the corresponding values for the signal significance and the signal over background ratio. Note that the MVA is always trained to the inclusive background sample, though differences in the kinematic distributions of the 4*b* and 2*b*2*j* processes are moderate; see Fig. 14. From Table 9 one observes that all categories exhibit a marked improvement from eliminating the contamination from light and charm jet mis-identification. For instance, in the intermediate category, $S/\sqrt{B}$ increases from 2.3 to 3.3 (1.9 to 2.9) in the no PU (PU80) case, with similar improvements in the resolved and boosted categories.

**Table 9:** Post-MVA number of signal and background events with ${\mathcal {L}}=3$ ab$^{-1}$. For the backgrounds, both the total number, $N_\mathrm{ev}^\mathrm{tot}$, and the 4*b* component only, $N_\mathrm{ev}^\mathrm{4b}$, are shown. Also provided are the values of the signal significance and the signal over background ratio, both separated in categories and for their combination. We quote the results without PU and for PU80+SK+Trim

Category	Signal	Background		$S/\sqrt{B_\mathrm{tot}}$	$S/\sqrt{B_\mathrm{4b}}$	$S/B_\mathrm{tot}$	$S/B_\mathrm{4b}$
	$N_\mathrm{ev}$	$N_\mathrm{ev}^\mathrm{tot}$	$N_\mathrm{ev}^\mathrm{4b}$				
*Boosted*							
No PU	290	$1.2\times 10^4$	$8.0\times 10^3$	2.7	3.2	0.03	0.04
PU80+SK+Trim	290	$3.7\times 10^4$	$1.2\times 10^4$	1.5	2.7	0.01	0.02
*Intermediate*							
No PU	130	$3.1\times 10^3$	$1.5\times 10^3$	2.3	3.3	0.04	0.08
PU80+SK+Trim	140	$5.6\times 10^3$	$2.4\times 10^3$	1.9	2.9	0.03	0.06
*Resolved*							
No PU	630	$1.1\times 10^5$	$5.8\times 10^4$	1.9	2.7	0.01	0.01
PU80+SK	640	$1.0\times 10^5$	$7.0\times 10^4$	2.0	2.6	0.01	0.01
*Combined*							
No PU				4.0	5.3		
PU80+SK+Trim				3.1	4.7		

In Table 9 we also provide the results for $S/\sqrt{B}$ obtained by combining the three categories. Taking into account all background components, we obtain for the case of $n_\mathrm{PU}=80$ an overall signal significance of

19 $$\begin{aligned} \left( \frac{S}{\sqrt{B}}\right) _\mathrm{tot} \simeq 3.1~(1.0),\quad {\mathcal {L}}=3000~(300)\,\mathrm{fb}^{-1}, \end{aligned}$$

indicating that a measurement of Higgs pair production in the $b\bar{b}b\bar{b}$ final state at the HL-LHC should be above the threshold for observation, even when realistic PU conditions are accounted for. A similar signal significance is obtained in the case of $n_\mathrm{PU}=150$. Under the assumption that the only relevant background would be the irreducible QCD 4*b* component, one obtains instead

20 $$\begin{aligned} \left( \frac{S}{\sqrt{B_\mathrm{4b}}}\right) _\mathrm{tot} \simeq 4.7~(1.5),\quad {\mathcal {L}}=3000~(300)\,\mathrm{fb}^{-1}. \end{aligned}$$

Therefore, a measurement of Higgs pair production in the $b\bar{b}b\bar{b}$ final state at the HL-LHC might be even above the threshold for discovery, provided the effects due to mis-identification of light and charm jets as *b*-jets can be reduced.

## Conclusions and outlook

In this work we have presented a feasibility study for the measurement of Higgs pair production in the $b\bar{b}b\bar{b}$ final state at the LHC. Our strategy is based on the combination of traditional cut-based analysis with state-of-the-art multivariate techniques. We take into account all relevant backgrounds, in particular the irreducible 4*b* and the reducible 2*b*2*j* and 4*j* QCD multijets. We have illustrated how the 2*b*2*j* component leads to a contribution comparable to that of QCD 4*b* production, due to a combination of parton shower effects, *b*-quark pair radiation, and selection requirements. We have also demonstrated the robustness of our analysis strategy under the addition of significant PU. In particular, we have explored two scenarios, $n_\mathrm{PU}=80$ and $n_\mathrm{PU}=150$, and we found a comparable overall signal significance in the two cases.

Combining the contributions from the resolved, intermediate and boosted categories, we find that, for ${\mathcal {L}}=3$ ab$^{-1}$, the signal significance for the production of Higgs pairs turns out to be $S/\sqrt{B}\simeq 3$. This indicates that, already from the $b\bar{b}b\bar{b}$ final state alone, it should be possible to claim observation of Higgs pair production at the HL-LHC. Our study also suggests possible avenues that the LHC experiments could explore to further improve this signal significance. One handle would be to reduce the contribution from light and charm jet mis-identification, ensuring that the irreducible 4*b* background dominates over the 2*b*2*j* component. This would allow one to enhance $S/\sqrt{B}$ almost to the discovery level; see Table 9. It would also be advantageous to improve the *b*-tagging efficiency, allowing to achieve higher signal yields. Another possibility would be to improve the mass resolution of the Higgs reconstruction in high-PU environments, and, more generally, to optimise the PU subtraction strategy in order to reduce the impact of PU in the modeling of kinematic variables and the associated degradation in the MVA discrimination.

Another challenging aspect of the measurement of Higgs pairs in the $b\bar{b}b\bar{b}$ final state is achieving an efficient triggering strategy. In order to reduce the rate from background QCD processes sufficiently, while being able to access the relevant $p_T$ regimes, (multi-)jet triggers using *b*-quark tagging information online for one or more jets are likely to be necessary. The additional rejection provided by these triggers could enable events to be selected efficiently, with four jets down to $p_T=40$ GeV in the resolved category, and boosted Higgs decays in large-*R* jets down to jet transverse momenta of $p_T=200$ GeV. In addition, good control of the multijet backgrounds and the experimental systematics of the MVA inputs will be important to achieve these sensitivities.

Our strategy relies on the modeling of the kinematic distributions of signal and background events, since these provide the inputs to the MVA discriminant. In this respect, it would be important, having established the key relevance of the $b\bar{b}b\bar{b}$ channel for the study of Higgs pair production, to revisit and improve the theoretical modeling of our signal and background simulation, in particular using NLO calculations matched to parton showers both for signal [17, 35] and for backgrounds [63, 76].

One important implication of this work is that it should be possible to significantly improve the accuracy on the extraction of the Higgs trilinear coupling $\lambda$ from a measurement of the $\sigma \left( hh\rightarrow b\bar{b}b\bar{b}\right)$ cross section, as compared to existing estimates. A determination of $\lambda$ in our approach is however rather non-trivial, involving not only generating signal samples for a wide range of values of $\lambda$, but also repeating the analysis optimisation, including the MVA training, for each of these values. This study is left to a future publication, where we will also compare the precision from the $b\bar{b}b\bar{b}$ final state with the corresponding precision that has been reported from other final states such as $b\bar{b}\gamma \gamma$ and $b\bar{b}\tau \tau$. It will also be interesting to perform this exercise for a 100 TeV hadron collider [11–14]. While at 100 TeV the signal yields would be increased, also the (gluon-driven) QCD multijet background would grow strongly. Revisiting the present analysis, including the MVA optimisation, at 100 TeV would also allow us to assess the accuracy of an extraction of the trilinear coupling $\lambda$ from the $b\bar{b}b\bar{b}$ final state at 100 TeV.

In this work we have considered only the SM production mechanism, but many BSM scenarios predict deviations in Higgs pair production, both at the level of total rates and of differential distributions. In the absence of new explicit degrees of freedom, deviations from the SM can be parametrised in the EFT framework using higher-order operators [14, 48]. Therefore, we plan to study the constraints on the coefficients of these effective operators that can be obtained from measurements of various kinematic distributions in the $hh\rightarrow b\bar{b}b\bar{b}$ process. Note that the higher rates of the $b\bar{b}b\bar{b}$ final state as compared to other final states, such as $b\bar{b}\gamma \gamma$, allow for better constraints upon operators that modify the high-energy behaviour of the theory, for instance, it would become possible to access the tail of the $m_{hh}$ distribution.

As in the case of the extraction of the Higgs trilinear coupling $\lambda$, such a study would be a computationally intensive task, since BSM dynamics will modify the shapes of the kinematic distributions and thus in principle each point in the EFT parameter space would require a re-optimisation with a newly trained MVA. In order to explore efficiently the BSM parameters without having to repeat the full analysis for each point, modern statistical techniques such as the Cluster Analysis method proposed in Ref. [46] might be helpful.

## Appendix A: Single Higgs backgrounds

As discussed in Sect. 2, in our analysis we neglect single Higgs production processes, since they are much smaller than both the signal and the main QCD multijet backgrounds. To explicitly demonstrate this, we have generated LO samples using MadGraph5_aMC@NLO for the following single-Higgs processes:

1. $$Z(\rightarrow b\bar{b})h(\rightarrow b\bar{b})$$ (electroweak)
2. $$t\bar{t}h(\rightarrow b\bar{b})$$
3. $$b\bar{b}h(\rightarrow b\bar{b})$$ (QCD)

For each processes, we have generated 1M events, and in Table 10 we list resulting the LO and NLO cross sections at the generation level. The subsequent decays and the corresponding branching fractions are not included in these cross sections, since these are taken care by the Pythia8 parton shower. The values of these branching fractions are listed in Table 11, corresponding to the most recent averages from the PDG. In the case of the $t\bar{t}h$ process, we consider only the fully hadronic decays of the top quark, since leptonic and semi-leptonic decays can be suppressed by means of a lepton veto.

**Table 10:** LO and NLO cross sections at the generation level for the single-Higgs background processes listed above, computed using MadGraph5_aMC@NLO. The subsequent decays and the corresponding branching fractions are not included in these generation-level cross sections

Sample	LO	NLO	*K*-factor
*Zh* (13 TeV)	$6.5 \times 10^{-1}$ pb	$7.7 \times 10^{-1}$ pb	1.19
$t\bar{t}h$ (13 TeV)	$3.8 \times 10^{-1}$ pb	$4.6 \times 10^{-1}$ pb	1.29
$b\bar{b}h$ (13 TeV)	$4.9 \times 10^{-1}$ pb	$6.1 \times 10^{-1}$ pb	1.22

**Table 11:** The values of the branching fractions applied to the single-Higgs background processes from Table 10, corresponding to the most updated PDG values

Sample	Decay	Branching fraction
*Zh*	($Z\rightarrow b\bar{b}$)($h\rightarrow b\bar{b}$)	0.086
$t\bar{t}h$	$(W\rightarrow q\bar{q})^2$($h\rightarrow b\bar{b}$)	0.26
$b\bar{h}h$	$h\rightarrow b\bar{b}$	0.57

**Table 12:** Signal and background cross sections at the end of the cut-based analysis (before the MVA is applied), in the case without PU. We separate the results into the three exclusive categories used in our analysis

	Sample	Pre-MVA cross section (fb)		
		Boosted	Intermediate	Resolved
Signal	$hh\rightarrow b\bar{b}b\bar{b}$	$3.5\times 10^{-1}$	$2.2\times 10^{-1}$	$1.2\times 10^{0}$
Backgrounds	QCD multijet	$2.5\times 10^{+2}$	$1.8\times 10^{+2}$	$4.9\times 10^{+3}$
	$Z(\rightarrow b\bar{b})h(\rightarrow b\bar{b})$	$2.0\times 10^{-2}$	$1.2\times 10^{-1}$	$7.5\times 10^{-1}$
	$t\bar{t}h(\rightarrow b\bar{b})$	$5.1\times 10^{-2}$	$6.3\times 10^{-3}$	$4.0\times 10^{-1}$
	$b\bar{b}h(\rightarrow b\bar{b})$	$2.3\times 10^{-3}$	$5.5\times 10^{-3}$	$2.6\times 10^{-1}$

In Table 12 we show the signal and background cross sections at the end of the cut-based analysis, before the MVA is applied, in the case without PU. We separate the results into the three exclusive categories used in our analysis. From this comparison, we see that as expected, at the end of the cut-based analysis, the single-Higgs backgrounds are smaller than the QCD multijet background by several orders of magnitude. In addition, we find that already at the end of the cut-based analysis the di-Higgs signal is also larger than all the single-Higgs backgrounds in all the selection categories. Since this discrimination can only be improved by the MVA, we conclude that neglecting single-Higgs backgrounds is a reasonable approximation. From Table 12 we also observe that in the resolved and intermediate categories $Zh\rightarrow b\bar{b}b\bar{b}$ is the dominant single-Higgs background, while $t\bar{t}h(\rightarrow b\bar{b})$ is instead the most important one in the boosted category.

## Footnotes

1 These techniques have also important applications in the subtraction of the UE/MPI contamination for jet reconstruction in heavy ion collisions [115].

2 This type of ANNs are the same as those used to parametrise Parton Distribution Functions in the NNPDF global analyses [121–124].

3 The impact of PU on the separate significance of the three categories exhibits some dependence on the specific choice for $n_\mathrm{PU}$ and on the settings of the PU subtraction strategy. We find, however, that the overall signal significance from combining the three categories is similar in the $n_\mathrm{PU}=80$ and $n_\mathrm{PU}=150$ cases.

## References

[1] ATLAS Collaboration, Physics at a high-luminosity LHC with ATLAS, in *Community Summer Study 2013: Snowmass on the Mississippi (CSS2013)*, Minneapolis, MN, USA, July 29-August 6, 2013. arXiv:1307.7292

[2] CMS Collaboration, Projected performance of an upgraded CMS detector at the LHC and HL-LHC: contribution to the snowmass process, in *Community Summer Study 2013: Snowmass on the Mississippi (CSS2013)*, Minneapolis, MN, USA, July 29-August 6, 2013. arXiv:1307.7135

[3] Baglio J, Djouadi A, Grober R, Muhlleitner M, Quevillon J (2013). The measurement of the Higgs self-coupling at the LHC: theoretical status. JHEP.

[4] Giudice G, Grojean C, Pomarol A, Rattazzi R (2007). The strongly-interacting light Higgs. JHEP.

[5] Contino R, Grojean C, Moretti M, Piccinini F, Rattazzi R (2010). Strong double Higgs production at the LHC. JHEP.

[6] ATLAS Collaboration, G. Aad et al., Searches for Higgs boson pair production in the $$hh\rightarrow bb\tau \tau , \gamma \gamma WW^*, \gamma \gamma bb, bbbb$$ channels with the ATLAS detector. Phys. Rev. D **92**, 092004 (2015). arXiv:1509.04670

[7] ATLAS Collaboration, G. Aad et al., Search for Higgs boson pair production in the $$b\bar{b}b\bar{b}$$ TeV with the ATLAS detector. Eur. Phys. J. C **75**(9), 412 (2015). arXiv:1506.00285 10.1140/epjc/s10052-015-3628-x PMC4564859 26380565

[8] ATLAS Collaboration, G. Aad et al., Search for Higgs boson pair production in the $$\gamma \gamma b\bar{b}$$ TeV from the ATLAS detector. Phys. Rev. Lett. **114**(8), 081802 (2015). arXiv:1406.5053 10.1103/PhysRevLett.114.081802 25768755

[9] CMS Collaboration, V. Khachatryan et al., Search for resonant pair production of Higgs bosons decaying to two bottom quark–antiquark pairs in proton–proton collisions at 8 TeV. Phys. Lett. B **749**, 560–582 (2015). arXiv:1503.04114

[10] CMS Collaboration, Search for the resonant production of two Higgs bosons in the final state with two photons and two bottom quarks. (2014). Report no. CMS-PAS-HIG-13-032

[11] N. Arkani-Hamed, T. Han, M. Mangano, L.-T. Wang, Physics opportunities of a 100 TeV proton–proton collider (2015). arXiv:1511.06495

[12] Barr AJ, Dolan MJ, Englert C, Ferreira de Lima DE, Spannowsky M (2015). Higgs self-coupling measurements at a 100 TeV hadron collider. JHEP.

[13] A. Papaefstathiou, Discovering Higgs boson pair production through rare final states at a 100 TeV collider. Phys. Rev. D **91**(11), 113016 (2015). arXiv:1504.04621

[14] A. Azatov, R. Contino, G. Panico, M. Son, Effective field theory analysis of double Higgs boson production via gluon fusion. Phys. Rev. D **92**(3), 035001 (2015). arXiv:1502.00539

[15] Contino R, Grojean C, Pappadopulo D, Rattazzi R, Thamm A (2014). Strong Higgs interactions at a linear collider. JHEP.

[16] LHC Higgs Cross Section Working Group, S. Dittmaier, C. Mariotti, G. Passarino, R. Tanaka (eds.), Handbook of LHC Higgs cross sections: 2. Differential distributions. CERN-2012-002 (CERN, Geneva, 2012). arXiv:1201.3084

[17] Frederix R, Frixione S, Hirschi V, Maltoni F, Mattelaer O, Torrielli P, Vryonidou E, Zaro M (2014). Higgs pair production at the LHC with NLO and parton-shower effects. Phys. Lett. B.

[18] de Florian D, Mazzitelli J (2013). Higgs boson pair production at next-to-next-to-leading order in QCD. Phys. Rev. Lett..

[19] D. de Florian, J. Mazzitelli, Higgs pair production at next-to-next-to-leading logarithmic accuracy at the LHC. JHEP **09**, 053 (2015). arXiv:1505.07122

[20] Baur U, Plehn T, Rainwater DL (2004). Probing the Higgs selfcoupling at hadron colliders using rare decays. Phys. Rev. D.

[21] V. Barger, L.L. Everett, C. B. Jackson, G. Shaughnessy, Higgs-pair production and measurement of the triscalar coupling at LHC(8,14). Phys. Lett. B **728**, 433–436 (2014). doi:10.1016/j.physletb.2013.12.013. arXiv:1311.2931

[22] C.T. Lu, J. Chang, K. Cheung, J.S. Lee, An exploratory study of Higgs-boson pair production. JHEP **1508**, 133 (2015). arXiv:1505.00957

[23] Baur U, Plehn T, Rainwater DL (2003). Examining the Higgs boson potential at lepton and hadron colliders: a comparative analysis. Phys. Rev. D.

[24] Barr AJ, Dolan MJ, Englert C, Spannowsky M (2014). Di-Higgs final states augMT2ed—selecting $$hh$$ events at the high luminosity LHC. Phys. Lett. B.

[25] Dolan MJ, Englert C, Spannowsky M (2012). Higgs self-coupling measurements at the LHC. JHEP.

[26] Dolan MJ, Englert C, Greiner N, Spannowsky M (2014). Further on up the road: $$hhjj$$ production at the LHC. Phys. Rev. Lett..

[27] Papaefstathiou A, Yang LL, Zurita J (2013). Higgs boson pair production at the LHC in the $$b \bar{b} W^+ W^-$$ channel. Phys. Rev. D.

[28] Wardrope D, Jansen E, Konstantinidis N, Cooper B, Falla R (2015). Non-resonant Higgs-pair production in the $$b\overline{b}$$ final state at the LHC. Eur. Phys. J. C.

[29] Ferreira de Lima DE, Papaefstathiou A, Spannowsky M (2014). Standard model Higgs boson pair production in the ($$b\overline{b}$$)($$ b\overline{b}$$) final state. JHEP.

[30] M. Slawinska, W. van den Wollenberg, B. van Eijk, S. Bentvelsen, Phenomenology of the trilinear Higgs coupling at proton–proton colliders. (2014). arXiv:1408.5010

[31] Chen C-R, Low I (2014). Double take on new physics in double Higgs boson production. Phys. Rev. D.

[32] Goertz F, Papaefstathiou A, Yang LL, Zurita J (2013). Higgs Boson self-coupling measurements using ratios of cross sections. JHEP.

[33] S. Dawson, A. Ismail, I. Low, What’s in the loop? The anatomy of double Higgs production. Phys. Rev. D **91**(11), 115008 (2015). arXiv:1504.05596

[34] Maltoni F, Vryonidou E, Zaro M (2014). Top-quark mass effects in double and triple Higgs production in gluon-gluon fusion at NLO. JHEP.

[35] Maierhfer P, Papaefstathiou A (2014). Higgs boson pair production merged to one jet. JHEP.

[36] Grigo J, Hoff J, Melnikov K, Steinhauser M (2013). On the Higgs boson pair production at the LHC. Nucl. Phys. B.

[37] Grigo J, Melnikov K, Steinhauser M (2014). Virtual corrections to Higgs boson pair production in the large top quark mass limit. Nucl. Phys. B.

[38] M.J. Dolan, C. Englert, N. Greiner, K. Nordstrom, M. Spannowsky, $$hhjj$$ production at the LHC. Eur. Phys. J. C **75**(8), 387 (2015). arXiv:1506.08008

[39] G. Brooijmans, R. Contino, B. Fuks, F. Moortgat, P. Richardson et al., Les Houches 2013: physics at TeV colliders: new physics working group report. (2014). arXiv:1405.1617

[40] Barger VD, Han T, Phillips RJN (1988). Double Higgs boson bremsstrahlung from $$W$$ and $$Z$$ bosons at supercolliders. Phys. Rev. D.

[41] Q.-H. Cao, Y. Liu, B. Yan, Measuring trilinear Higgs coupling in $$WHH$$ and $$ZHH$$ productions at the HL-LHC. (2015). arXiv:1511.03311

[42] Englert C, Krauss F, Spannowsky M, Thompson J (2015). Di-Higgs phenomenology in $$t\bar{t}hh$$: the forgotten channel. Phys. Lett. B.

[43] Ling L-S, Zhang R-Y, Ma W-G, Guo L, Li W-H (2014). NNLO QCD corrections to Higgs pair production via vector boson fusion at hadron colliders. Phys. Rev. D.

[44] Nishiwaki K, Niyogi S, Shivaji A (2014). $$ttH$$ Anomalous coupling in double Higgs production. JHEP.

[45] Q.H. Cao, B. Yan, D.M. Zhang, H. Zhang, Resolving the degeneracy in single Higgs production with Higgs pair production. Phys. Lett. B **752**, 285 (2016). arXiv:1508.06512

[46] A. Carvalho, M. Dall’Osso, T. Dorigo, F. Goertz, C.A. Gottardo, M. Tosi, Higgs pair production: choosing benchmarks with cluster analysis. JHEP **04**, 126 (2016). doi:10.1007/JHEP04(2016)126. arXiv:1507.02245

[47] Liu N, Hu S, Yang B, Han J (2015). Impact of top-Higgs couplings on di-Higgs production at future colliders. JHEP.

[48] Goertz F, Papaefstathiou A, Yang LL, Zurita J (2015). Higgs boson pair production in the D = 6 extension of the SM. JHEP.

[49] H.-J. He, J. Ren, W. Yao, Probing new physics of cubic Higgs boson interaction via Higgs pair production at hadron colliders. Phys. Rev. D **93**, 015003 (2016). doi:10.1103/PhysRevD.93.015003. arXiv:1506.03302

[50] R. Grober, M. Muhlleitner, M. Spira, J. Streicher, NLO QCD corrections to Higgs pair production including dimension-6 operators. JHEP **09**, 092 (2015). arXiv:1504.06577

[51] Gouzevitch M, Oliveira A, Rojo J, Rosenfeld R, Salam GP (2013). Scale-invariant resonance tagging in multijet events and new physics in Higgs pair production. JHEP.

[52] Cooper B, Konstantinidis N, Lambourne L, Wardrope D (2013). Boosted $$hh b\overline{b}b\overline{b}$$: a new topology in searches for TeV-scale resonances at the LHC. Phys. Rev. D.

[53] No JM, Ramsey-Musolf M (2014). Probing the Higgs portal at the LHC through resonant di-Higgs production. Phys. Rev. D.

[54] Z. Wen-Juan, M. Wen-Gan, Z. Ren-You, L. Xiao-Zhou, G. Lei, C. Chong, Double Higgs boson production and decay in Randall–Sundrum model at hadron colliders. Phys. Rev. D **92**(1), 116005 (2015). doi:10.1103/PhysRevD.92.116005. arXiv:1512.01766

[55] A. Belyaev, M. Drees, O.J.P. Eboli, J.K. Mizukoshi, S.F. Novaes, Supersymmetric Higgs pair discovery prospects at hadron colliders, in *Proceedings, International Europhysics Conference on High energy physics (EPS-HEP 1999)*, pp. 748–751. arXiv:hep-ph/9910400

[56] Han C, Ji X, Wu L, Wu P, Yang JM (2014). Higgs pair production with SUSY QCD correction: revisited under current experimental constraints. JHEP.

[57] Hespel B, Lopez-Val D, Vryonidou E (2014). Higgs pair production via gluon fusion in the two-Higgs-doublet model. JHEP.

[58] L. Wu, J.M. Yang, C.-P. Yuan, M. Zhang, Higgs self-coupling in the MSSM and NMSSM after the LHC Run 1. Phys. Lett. B **747**, 378–389 (2015). arXiv:1504.06932

[59] Cao J, Li D, Shang L, Wu P, Zhang Y (2014). Exploring the Higgs sector of a most natural NMSSM and its prediction on Higgs pair production at the LHC. JHEP.

[60] Ellwanger U (2013). Higgs pair production in the NMSSM at the LHC. JHEP.

[61] Cao J, Heng Z, Shang L, Wan P, Yang JM (2013). Pair production of a 125 GeV Higgs boson in MSSM and NMSSM at the LHC. JHEP.

[62] CMS Collaboration, V. Khachatryan et al., Searches for a heavy scalar boson $${\rm H}$$ decaying to a pair of 125 GeV Higgs bosons hh or for a heavy pseudoscalar boson A decaying to Zh, in the final states with h to tau tau. Phys. Lett. B **755**, 217–244 (2016). doi:10.1016/j.physletb.2016.01.056. arXiv:1510.01181

[63] Alwall J, Frederix R, Frixione S, Hirschi V, Maltoni F (2014). The automated computation of tree-level and next-to-leading order differential cross sections, and their matching to parton shower simulations. JHEP.

[64] Plehn T, Spira M, Zerwas P (1996). Pair production of neutral Higgs particles in gluon–gluon collisions. Nucl. Phys. B.

[65] V. Hirschi, O. Mattelaer, Automated event generation for loop-induced processes. JHEP **10**, 146 (2015). arXiv:1507.00020

[66] NNPDF Collaboration, R.D. Ball et al., Parton distributions for the LHC Run II. JHEP **1504**, 040 (2015). arXiv:1410.8849

[67] Buckley A, Ferrando J, Lloyd S, Nordstrm K, Page B (2015). LHAPDF6: parton density access in the LHC precision era. Eur. Phys. J. C.

[68] ATLAS Collaboration, G. Aad et al., Measurement of the Higgs boson mass from the $$H\rightarrow \gamma \gamma $$ collision data. Phys. Rev. D **90**(5), 052004 (2014). arXiv:1406.3827

[69] CMS Collaboration, V. Khachatryan et al., Precise determination of the mass of the Higgs boson and tests of compatibility of its couplings with the standard model predictions using proton collisions at 7 and 8 TeV. Eur. Phys. J. C **75**(5), 212 (2015). arXiv:1412.8662 10.1140/epjc/s10052-015-3351-7 PMC4433454 25999783

[70] ATLAS, CMS Collaboration, G. Aad et al., Combined measurement of the Higgs boson mass in $$pp$$ and 8 TeV with the ATLAS and CMS experiments. Phys. Rev. Lett. **114**, 191803 (2015). arXiv:1503.07589 10.1103/PhysRevLett.114.191803 26024162

[71] Sjostrand T, Mrenna S, Skands PZ, Brief A (2008). Introduction to PYTHIA 8.1. Comput. Phys. Commun..

[72] Sjstrand T, Ask S, Christiansen JR, Corke R, Desai N (2015). An introduction to PYTHIA 8.2. Comput. Phys. Commun..

[73] Skands P, Carrazza S, Rojo J (2014). Tuning PYTHIA 8.1: the Monash 2013 tune. Eur. Phys. J. C.

[74] Ball RD, Bertone V, Carrazza S, Deans CS, Del Debbio L (2013). Parton distributions with LHC data. Nucl. Phys. B.

[75] NNPDF Collaboration, R.D. Ball, V. Bertone, S. Carrazza, L. Del Debbio, S. Forte, A. Guffanti, N.P. Hartland, J. Rojo, Parton distributions with QED corrections. Nucl. Phys. B **877**, 290–320 (2013). arXiv:1308.0598

[76] T. Gleisberg et al., Event generation with SHERPA 1.1. JHEP **02**, 007 (2009). arXiv:0811.4622

[77] Bern Z, Diana G, Dixon L, Febres F (2012). Cordero, S. Hoeche et al., Four-jet production at the large hadron collider at next-to-leading order in QCD. Phys. Rev. Lett..

[78] Czakon M, Fiedler P, Mitov A (2013). Total top-quark pair-production cross section at hadron colliders through $$O(\frac{4}{S})$$. Phys. Rev. Lett..

[79] ATLAS Collaboration, G. Aad et al., Search for the Standard Model Higgs boson produced in association with a vector boson and decaying to a $$b$$-quark pair with the ATLAS detector. Phys. Lett. B **718**, 369–390 (2012). arXiv:1207.0210

[80] CMS Collaboration, S. Chatrchyan et al., Search for the standard model Higgs boson produced in association with a W or a Z boson and decaying to bottom quarks. Phys. Rev. D **89**(1), 012003 (2014). arXiv:1310.3687

[81] ATLAS Collaboration, G. Aad et al., Search for the $$b\bar{b}$$ production with the ATLAS detector. JHEP **01**, 069 (2015). arXiv:1409.6212

[82] Cacciari M, Salam GP, Soyez G (2012). FastJet user manual. Eur. Phys. J. C.

[83] Cacciari M, Salam GP (2006). Dispelling the $$N^{3}$$ myth for the $$k_t$$ jet-finder. Phys. Lett. B.

[84] Cacciari M, Salam GP, Soyez G (2008). The anti-k(t) jet clustering algorithm. JHEP.

[85] ATLAS Collaboration, G. Aad et al., Jet energy measurement and its systematic uncertainty in proton–proton collisions at $$\sqrt{s}=7$$ TeV with the ATLAS detector. Eur. Phys. J. C **75**(1), 17 (2015). arXiv:1406.0076 10.1140/epjc/s10052-014-3190-y PMC4684939 26709345

[86] ATLAS Collaboration, Performance of large-R jets and jet substructure reconstruction with the ATLAS detector. (2012). Report no. ATLAS-CONF-2012-065

[87] Butterworth JM, Davison AR, Rubin M, Salam GP (2008). Jet substructure as a new Higgs search channel at the LHC. Phys. Rev. Lett..

[88] Dokshitzer YL, Leder G, Moretti S, Webber B (1997). Better jet clustering algorithms. JHEP.

[89] M. Wobisch, T. Wengler, Hadronization corrections to jet cross-sections in deep inelastic scattering, in *Monte Carlo generators for HERA physics. Proceedings, Workshop, Hamburg, Germany*, 1998-1999 (1998). arXiv:hep-ph/9907280

[90] Salam GP (2010). Towards jetography. Eur. Phys. J. C.

[91] ATLAS Collaboration, G. Aad et al., Performance of jet substructure techniques for large-$$R$$ = 7 TeV using the ATLAS detector. JHEP **1309**, 076 (2013). arXiv:1306.4945

[92] Butterworth J, Cox B, Forshaw JR (2002). $$W W$$ scattering at the CERN LHC. Phys. Rev. D.

[93] Ellis SD, Soper DE (1993). Successive combination jet algorithm for hadron collisions. Phys. Rev. D.

[94] Thaler J, Van Tilburg K (2011). Identifying boosted objects with N-subjettiness. JHEP.

[95] Thaler J, Van Tilburg K (2012). Maximizing boosted top identification by minimizing N-subjettiness. JHEP.

[96] Catani S, Dokshitzer YL, Seymour MH, Webber BR (1993). Longitudinally invariant $$K_t$$ clustering algorithms for hadron hadron collisions. Nucl. Phys. B.

[97] Larkoski AJ, Salam GP, Thaler J (2013). Energy correlation functions for jet substructure. JHEP.

[98] Larkoski AJ, Moult I, Neill D (2014). Power counting to better jet observables. JHEP.

[99] ATLAS Collaboration, G. Aad et al., Performance of $$b$$-jet identification in the ATLAS experiment. JINST **11**(04), P04008 (2016). doi:10.1088/1748-0221/11/04/P04008. arXiv:1512.01094

[100] CMS Collaboration, V. Khachatryan et al., Measurement of $$B\bar{B}$$ angular correlations based on secondary vertex reconstruction at $$\sqrt{s}=7$$ TeV. JHEP **1103**, 136 (2011). arXiv:1102.3194

[101] CMS Collaboration, S. Chatrchyan et al., Identification of b-quark jets with the CMS experiment. JINST **8**, P04013 (2013). arXiv:1211.4462

[102] Cacciari M, Salam GP (2008). Pileup subtraction using jet areas. Phys. Lett. B.

[103] ATLAS Collaboration, Calibration of *b*-tagging using dileptonic top pair events in a combinatorial likelihood approach with the ATLAS experiment. (2014). Report no. ATLAS-CONF-2014-004

[104] Cacciari M, Salam GP, Sapeta S (2010). On the characterisation of the underlying event. JHEP.

[105] ATLAS Collaboration, Pile-up subtraction and suppression for jets in ATLAS. (2013). Report no. ATLAS-CONF-2013-083

[106] Krohn D, Thaler J, Wang L-T (2010). Jet trimming. JHEP.

[107] Krohn D, Schwartz MD, Low M, Wang L-T (2014). Jet cleansing: pileup removal at high luminosity. Phys. Rev. D.

[108] Cacciari M, Rojo J, Salam GP, Soyez G (2008). Quantifying the performance of jet definitions for kinematic reconstruction at the LHC. JHEP.

[109] Ellis SD, Vermilion CK, Walsh JR (2010). Recombination algorithms and jet substructure: pruning as a tool for heavy particle searches. Phys. Rev. D.

[110] Bertolini D, Harris P, Low M, Tran N (2014). Pileup per particle identification. JHEP.

[111] Cacciari M, Salam GP, Soyez G (2015). SoftKiller, a particle-level pileup removal method. Eur. Phys. J. C.

[112] Cacciari M, Salam GP, Soyez G (2015). Use of charged-track information to subtract neutral pileup. Phys. Rev. D.

[113] Berta P, Spousta M, Miller DW, Leitner R (2014). Particle-level pileup subtraction for jets and jet shapes. JHEP.

[114] Larkoski AJ, Marzani S, Soyez G, Thaler J (2014). Soft drop. JHEP.

[115] Cacciari M, Rojo J, Salam GP, Soyez G (2011). Jet reconstruction in heavy ion collisions. Eur. Phys. J. C.

[116] Soper DE, Spannowsky M (2011). Finding physics signals with shower deconstruction. Phys. Rev. D.

[117] Soper DE, Spannowsky M (2013). Finding top quarks with shower deconstruction. Phys. Rev. D.

[118] Baldi P, Sadowski P, Whiteson D (2015). Enhanced Higgs boson to $$\tau ^+\tau ^-$$ search with deep learning. Phys. Rev. Lett..

[119] CDF and D0 Collaboration, T. Aaltonen et al., Evidence for a particle produced in association with weak bosons and decaying to a bottom–antibottom quark pair in Higgs boson searches at the Tevatron. Phys. Rev. Lett. **109**, 071804 (2012). arXiv:1207.6436 10.1103/PhysRevLett.109.071804 23006359

[120] Z. Kang, P. Ko, J. Li, New avenues to heavy right-handed neutrinos with pair production at hadronic colliders. Phys. Rev. D **93**(7), 075037 (2016). doi:10.1103/PhysRevD.93.075037. arXiv:1512.08373

[121] The NNPDF Collaboration, L. Del Debbio, S. Forte, J.I. Latorre, A. Piccione, J. Rojo, Unbiased determination of the proton structure function f2(p) with faithful uncertainty estimation. JHEP **03**, 080 (2005). arXiv:hep-ph/0501067

[122] The NNPDF Collaboration, R.D. Ball et al., A determination of parton distributions with faithful uncertainty estimation. Nucl. Phys. B **809**, 1–63 (2009). arXiv:0808.1231

[123] The NNPDF Collaboration, R.D. Ball et al., Impact of heavy quark masses on parton distributions and LHC phenomenology. Nucl. Phys. B **849**, 296–363 (2011). arXiv:1101.1300

[124] The NNPDF Collaboration, R.D. Ball et al., A first unbiased global NLO determination of parton distributions and their uncertainties. Nucl. Phys. B **838**, 136–206 (2010). arXiv:1002.4407

[125] Allanach BC, Grellscheid D, Quevedo F (2004). Genetic algorithms and experimental discrimination of SUSY models. JHEP.

[126] Rojo J, Latorre JI (2004). Neural network parametrization of spectral functions from hadronic tau decays and determination of qcd vacuum condensates. JHEP.

[127] Abel S, Rizos J (2014). Genetic algorithms and the search for viable string vacua. JHEP.

[128] Nesseris S, Garcia-Bellido J (2012). A new perspective on dark energy modeling via genetic algorithms. JCAP.

